# Effect of High Viscosity on Energy Metabolism and Kinematics of Spermatozoa from Three Mouse Species Incubated under Capacitating Conditions

**DOI:** 10.3390/ijms232315247

**Published:** 2022-12-03

**Authors:** Ana Sanchez-Rodriguez, Ester Sansegundo, Maximiliano Tourmente, Eduardo R. S. Roldan

**Affiliations:** 1 Departmento de Biodiversidad y Biología Evolutiva, Museo Nacional de Ciencias Naturales (CSIC), 28006 Madrid, Spain; 2 Centro de Biología Celular y Molecular, Facultad de Ciencias Exactas, Físicas y Naturales, Universidad Nacional de Córdoba (FCEFyN—UNC), Córdoba X5016GCA, Argentina; 3 Instituto de Investigaciones Biológicas y Tecnológicas, Consejo Nacional de Investigaciones Científicas y Técnicas (IIByT—CONICET, UNC), Córdoba X5016GCA, Argentina

**Keywords:** capacitation, hyperactivation, sperm motility, bioenergetics, ATP

## Abstract

In order to sustain motility and prepare for fertilization, sperm require energy. The characterization of sperm ATP production and usage in mouse species revealed substantial differences in metabolic pathways that can be differentially affected by capacitation. Moreover, spermatozoa encounter different environments with varying viscoelastic properties in the female reproductive tract. Here, we examine whether viscosity affects sperm ATP levels and kinematics during capacitation in vitro. Sperm from three mouse species (*Mus musculus*, *M. spretus*, *M. spicilegus*) were incubated under capacitating conditions in a modified Tyrode’s medium containing bicarbonate, glucose, pyruvate, lactate, and bovine serum albumin (mT-BH) or in a bicarbonate-free medium as a non-capacitating control. Viscosity was increased with the inclusion of polyvinylpyrrolidone. ATP was measured with a bioluminescence kit, and kinematics were examined with a computer-aided sperm analysis system. In *M. musculus* sperm, ATP declined during capacitation, but no differences were found between non-capacitating and capacitating sperm. In contrast, in *M. spretus* and *M. spicilegus*, ATP levels decreased in capacitating sperm. Increasing viscosity in the medium did not modify the timing or proportion of cells undergoing capacitation but did result in additional time- and concentration-dependent decreases in ATP in *M. spretus* and *M. spicilegus* under capacitating conditions. Additionally, increased viscosity altered both velocity and trajectory descriptors. The limited impact of capacitation and higher viscosity on *M. musculus* sperm ATP and kinematics could be related to the low intensity of postcopulatory sexual selection in this species. Responses seen in the other two species could be linked to the ability of their sperm to perform better under enhanced selective pressures.

## 1. Introduction 

Mammalian spermatozoa undergo a process of maturation in the female reproductive tract known as “capacitation” [1], which enables them to interact with and fertilize the ovum [2,3,4]. A consequence of this process is the development of a particular type of motility, with a characteristic pattern known as “hyperactivation” [5]. In aqueous media with low viscosity, such as those used for in vitro studies, the hyperactivated movement has an asymmetric pattern, with a vigorous and high-frequency flagellar beat, and spermatozoa are less progressive [6,7]. In fluids of the female tract, this movement is thought to allow sperm cells to swim forward, improving progression in a viscoelastic medium [8,9]. Viscoelastic fluids may also promote collective sperm swimming [10,11,12]. The viscosity of fluids in the female reproductive tract is not uniform nor homogeneous; it varies among different tract regions and during the estrous cycle [13,14,15]. Hyperactivation also enables sperm to respond to chemical stimuli (chemotaxis) [16], temperature changes (thermotaxis) [17], and fluid flow (rheotaxis) [15,18]. In high viscosity, spermatozoa appear to respond better to chemotactic signals, such as those of progesterone [19].

Sperm motility relies on ATP availability, and it consumes about 70% of the ATP produced by the cell [20]. A clear association exists between sperm ATP content and sperm motility, both within [21,22,23] and between species [24,25]. Spermatozoa also require an ATP supply to sustain essential cellular processes such as the maintenance of cell integrity and cell signaling [26,27,28]. In mammals, the majority of sperm ATP is produced through two metabolic pathways that are localized to two different regions of this highly compartmentalized cell [29,30]: oxidative phosphorylation (OXPHOS) that occurs in the midpiece’s mitochondria and glycolysis that occurs in the fibrous sheath of the flagellum’s principal piece. Although the exogenous metabolic substrates used for both pathways and the oxygen needed for oxidative phosphorylation appear to be abundant in the female reproductive tract [31,32], a positive balance between ATP production and consumption is crucial for sperm to maintain high-performance levels over extended periods. The physiological changes that underlie capacitation, such as the activation of phospholipases or protein phosphorylation, may influence sperm’s energetic balance by altering ATP production, consumption, or both [33,34].

The production of ATP during sperm capacitation is not yet fully understood. Recent studies have revealed a decrease in ATP levels during sperm capacitation in three mouse species [34], with species-specific differences that may relate to their different energetic budget. One species (*M. musculus*) had a decline in ATP during capacitation that was not different from that observed in non-capacitating cells, whereas in two other species (*M. spretus*, *M. spicilegus*), the decline in ATP levels was much higher under capacitating conditions than in non-capacitating controls [34]. The bioenergetics of murine sperm capacitation involves changes in both glycolytic and respiratory pathways. Studies using extracellular flux analysis revealed variations in the glycolysis and OXPHOS rates as a consequence of capacitation [35,36,37,38,39]. However, there are still conflicting results regarding the extent and direction of these changes and the functional role of each metabolic pathway. Consumption of ATP in *M. musculus* sperm does not appear to be enhanced in capacitating sperm in relation to non-capacitating ones [39]. Thus, the ATP balance between production and consumption may vary between species and the ATP levels seen in the cells under different conditions may not be consistent.

There is limited information on the effects of viscosity on sperm bioenergetics. In invertebrate spermatozoa, higher viscosity alters sperm movement [40,41] and the effects of viscosity are further influenced by ATP concentration [42]. In sea urchins, for instance, increasing the viscosity of the aqueous medium led to modified beating waveforms of the axonemes and to higher energy consumption per beat cycle [43]. 

To further understand sperm bioenergetics during processes that take place in preparation for fertilization, we examined if changes in viscosity influence ATP content in the spermatozoa of three mouse species (*M. musculus*, *M. spretus*, *M. spicilegus*) with different bioenergetic patterns and sperm kinematics. Restrictions on sperm movement imposed by high viscosity may promote an increase in ATP demand, thereby causing a decrease in ATP levels (net balance) in sperm. We hypothesized that increases in viscosity would correlate negatively with ATP content if sperm required more energy to sustain forward motility in more viscous media. Furthermore, extended incubation times under high viscosity would result in lower ATP content. Finally, if capacitation enhances ATP demands in spermatozoa, capacitating spermatozoa incubated in a highly viscous medium may exhibit further declines in ATP content. Since spermatozoa from the three species differ in mechanisms for ATP production and also vary in how they consume ATP, we also speculated that there would be an association between these factors and ATP balance and that this would be reflected in kinematic parameters.

## 2. Results

### 2.1. Effect of High Viscosity on Capacitation

Spermatozoa were incubated under non-capacitating (mT-H) or capacitating (mT-BH) media, and the percentage of sperm exhibiting a “B pattern”, indicative of capacitation, was assessed by staining with Hoechst 33258 and chlortetracycline (see Materials and Methods). The proportion of capacitated cells identified with this method correlates well with that identified using other methods that assess capacitation, such as protein phosphorylation, spontaneous or zona-pellucida-induced acrosome reaction, fertilization, and pronuclear formation [44,45]. 

**Figure 1:**
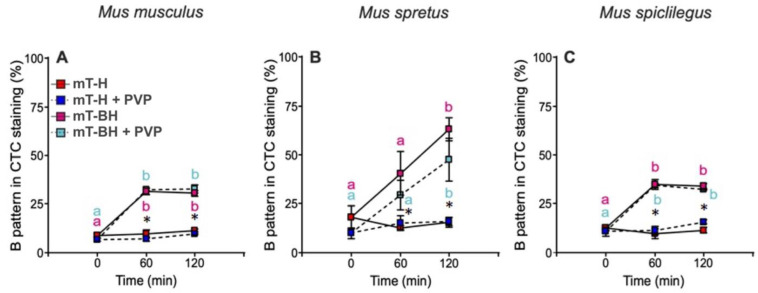
Percentage of spermatozoa exhibiting pattern B after CTC staining in the spermatozoa of three mouse species (*M. musculus*, *M. spretus*, and *M. spicilegus*) over time when incubated in mT-H, mT-H + PVP, mT-BH, and mT-BH + PVP. Data are means ± SE (*M. musculus*: N = 4; *M. spretus*: N = 5; *M. spicilegus*: N = 4). (**A**), *M. musculus*. (**B**), *M. spretus*. (**C**), *M. spicilegus*. Red squares: non-capacitating conditions with low viscosity, mT−H medium under air. Pink squares and letters: capacitating conditions with high viscosity, mT−BH + PVP medium under air. Dark blue squares: non-capacitating conditions with low viscosity, mT−BH medium under 5% CO_2_/air. Light blue squares and letters: capacitating conditions with high viscosity, mT−BH + PVP under 5% CO_2_/air. Different letters indicate significant differences between times for the same treatment in a Fisher post-hoc test (*p* < 0.05). Asterisks indicate significant differences between non-capacitating and capacitating conditions (*p* < 0.0001). There were no differences associated with the viscosity of the medium in non-capacitating or capacitating conditions.

When sperm from three mouse species (*Mus musculus*, *M. spretus*, *M. spicilegus*) were incubated in mT-H, only a low percentage of cells exhibited pattern B, which remained low over time (9–12%), up to 2 h after the start of incubation (Figure 1). On the other hand, when sperm were incubated in medium containing bicarbonate (mT-BH), spermatozoa from the three mouse species exhibited a significant time-dependent increase in the proportion of B cells (Figure 1). Under these conditions, the values reached a maximum at 60 min in *M. musculus* (32%) and *M. spicilegus* (38%) but continued rising until the end of incubation, at 120 min, in *M. spretus* (53%).

The addition of 2% polyvinylpyrrolidone (PVP) to increase the viscosity of the media resulted in a low proportion of sperm cells with a B pattern when they were incubated under non-capacitating conditions for up to 120 min (Figure 1). The proportion of cells under these conditions was not different from that recorded in the absence of PVP (Figure 1). In sperm incubated under capacitating conditions (mT-BH) in the presence of 2% PVP, a rise in the proportion of B pattern cells was observed, which was not different from that observed in cells incubated in its absence (Figure 1).

The viability of spermatozoa remained high under all conditions of incubation, regardless of the absence or presence of 2% PVP (Figure S1). In two species (*M. spretus*, *M. spicilegus*), there seemed to be a higher proportion of viable cells towards the end of incubation, but this may be due to slight sampling differences; in any case, the proportion of viable cells was consistently high throughout. The proportion of motile sperm showed some decline over time in the three species (Figure S1). There were no differences due to incubation under non-capacitating or capacitating conditions. Similarly, there were no differences in the percentage of motile sperm between cells incubated in the absence or the presence of 2% PVP (Figure S1).

### 2.2. Effect of Different Viscosities on Sperm Kinematics and ATP Content 

To assess the effects of viscosity on sperm kinematics, non-capacitating and capacitating spermatozoa were exposed to 0, 1, 2, and 4% PVP. Samples were taken at 30 min (for sperm kinematics) or at 0, 15, and 30 min (for ATP levels). These sampling times were chosen to avoid the complete cancellation of motility that may take place at high PVP concentrations [19].

#### 2.2.1. Effect of Different Viscosities on Sperm Kinematics

When spermatozoa were incubated in non-capacitating conditions, increases in the concentration of PVP to up to 4% led to different results in the three species (Table 1, Table 2 and Table 3). In *M. musculus*, there was a slight but significant decrease in VCL with 4% PVP in comparison to the control (0% PVP), but none of the other kinematic parameters exhibited differences when sperm were incubated in varying concentrations of PVP (Table 1). On the other hand, in *M. spretus*, increases in PVP concentrations resulted in a significant concentration-dependent decline in VCL, VSL, VAP, and ALH (Table 2). In *M. spicilegus*, there was also a concentration-dependent decrease in velocities, STR, and BCF but not in ALH (Table 3). 

For sperm cells in capacitating conditions (Table 1, Table 2 and Table 3), results showed that *M. musculus* spermatozoa were not affected by increasing viscosity because sperm kinematic values were similar across PVP concentrations (Table 1). Spermatozoa from *M. spretus* were influenced by viscosity because there was a decrease in velocity (VCL, VSL, VAP) and in STR and ALH with increasing concentrations of PVP in the medium (Table 2). Finally, in *M. spicilegus* there was a concentration-dependent effect on sperm velocity (VCL, VSL, VAP) and also in ALH (Table 3).

#### 2.2.2. Effect of Different Viscosities on ATP Levels

In spermatozoa incubated in non-capacitating conditions, increases in the concentration of PVP to up to 4% did not appear to have any effect on ATP levels in *M. musculus* spermatozoa because similar ATP values were seen across the range of viscosities and between 0 and 30 min (Table 4). In spermatozoa from *M. spretus*, there were no significant differences in ATP values between 0–4% PVP at each time point (0, 15, or 30 min), but there were differences between incubation times. For *M. spicilegus* spermatozoa, no differences in ATP values were observed at 0 min with increasing concentrations of PVP. At 15 and 30 min of incubation, there was a trend toward lower ATP values at 4% PVP (*p* < 0.1) in comparison to 0% PVP. 

Under capacitating conditions, increasing PVP concentrations did not appear to have any effect on ATP values in *M. musculus* at any incubation time. In contrast, in *M. spretus* and *M. spicilegus*, there was a decrease in ATP levels with increasing concentrations of PVP, particularly at 30 min of incubation (Table 4).

#### 2.2.3. Relationship between ATP Levels and Sperm Kinematics

**Figure 2:**
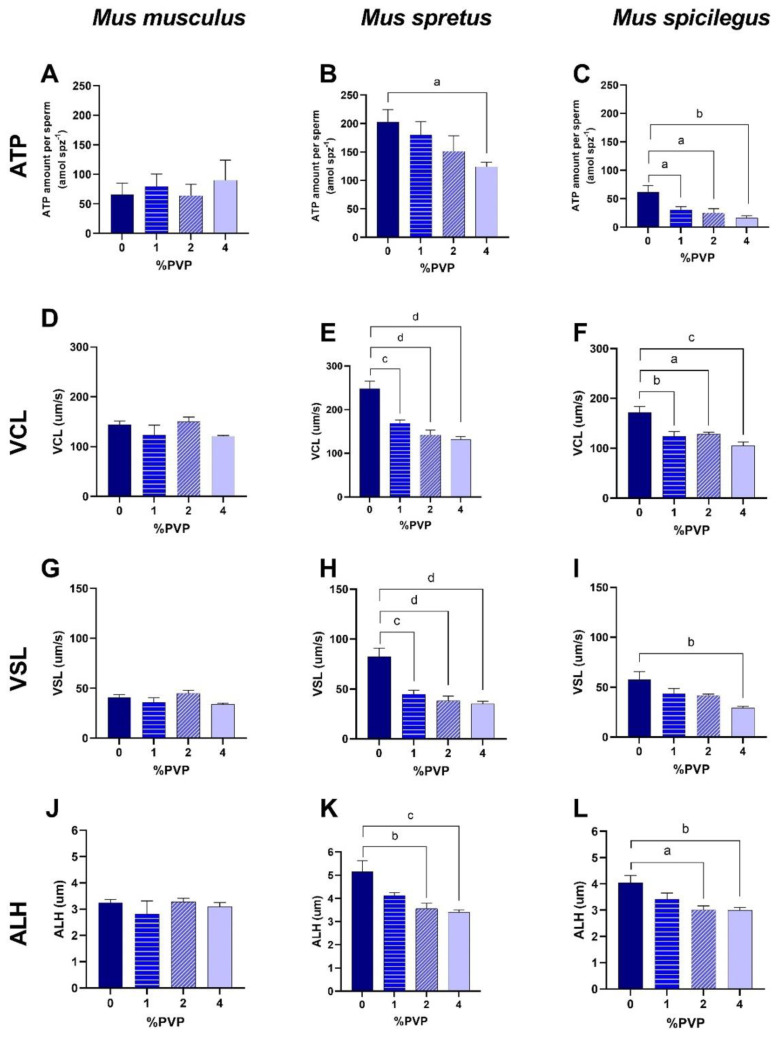
Variation in ATP levels and kinematics in spermatozoa from three mouse species incubated under capacitating conditions with increasing concentrations of PVP. Sperm were incubated in mT-BH medium in 5% CO_2_/air with 0, 1, 2, or 4% PVP for 30 min. Data are means ± SE (*M. musculus*: N = 4; *M. spretus*: N = 7; *M. spicilegus*: N = 7). (**A**–**C**): Amount of ATP per sperm (amol spz^−1^). (**D**–**F**): curvilinear velocity (VCL). (**G**–**I**): straight-line velocity (VSL). (**J**–**L**): amplitude of lateral head displacement (ALH). (**A**,**D**,**G**,**J**): *Mus musculus.* (**B**,**E**,**H**,**K**): *Mus spretus.* (**C**,**F**,**I**,**L**): *Mus spicilegus.* Letters indicate statistically significant differences (a: *p* < 0.05; b: *p* < 0.01; c: *p* < 0.001; d: *p* < 0.0001) between PVP concentrations and control (0% PVP) in Tukey’s post-hoc test.

To understand if differences in ATP levels were related to changes in sperm swimming parameters, results from the quantification of ATP values and sperm kinematics were compared. Analyses were carried out on sperm incubated under capacitating conditions (mT-BH in 5% CO_2_/air) for 30 min. As can be seen in Figure 2, no changes were observed in ATP levels in *M. musculus* sperm when incubated in capacitating medium with up to 4% PVP. Similarly, no significant changes were found in VCL, VSL, or ALH with varying concentrations of PVP. In contrast, *M. spretus* exhibited a decrease in ATP levels with 4% PVP, whereas *M. spicilegus* spermatozoa experienced a concentration-dependent decline in ATP values with 1–4% PVP. In parallel, values of VCL, VSL, and ALH showed a concentration-dependent decrease as % PVP was augmented in the incubation medium. These results indicate that increases in viscosity in the medium lead to a lowering of ATP values and a decrease in sperm kinematics parameters, which suggests that higher viscosity demands more energy and that, even with more ATP use (decrease), sperm still experience reduced motion. 

### 2.3. Effect of High Viscosity on Sperm Kinematics and ATP Content at Different Times of Incubation 

Spermatozoa were incubated in mT-H or mT-BH with 2% PVP for up to 120 min, and samples were taken at 0, 60, and 120 min for sperm kinematics and ATP analyses.

#### 2.3.1. Effect of High Viscosity on Kinematics at Different Times of Incubation

Since sperm swimming parameters are highly correlated [46], principal component analysis (PCA) was performed to obtain variables that synthesize information on sperm kinetics for each species and, thus, improve the analyses of results obtained after incubation under different conditions. Two sets of variables were analyzed, namely, overall sperm velocity (OSV) and overall trajectory shape (OTS). OSV was estimated using curvilinear velocity (VCL), straight-line velocity (VSL), and average path velocity (VAP). Analyses revealed that the first principal component (OSV 1) accounted for 94.2% of the total variability in *M. musculus*, 97.6% in *M. spretus*, and 92.2% in *M. spicilegus*; thus, this component was regarded as a representative measure of sperm velocity (Figure S2; Tables S1–S3). OTS was constructed using linearity (LIN), straightness (STR), the wobble coefficient (WOB), the amplitude of lateral head displacement (ALH), and beat cross frequency (BCF). The first principal component (OTS 1) represented 64.5% of the total variability in *M. musculus*, 49.4% in *M. spretus*, and 65.8% in *M. spicilegus*. A second principal component (OTS 2) represented 26.2% of the variability in *M. musculus*, 37.2% in *M. spretus*, and 27.8% in *M. spicilegus*. Both principal components, OTS 1 and OTS2, were used to describe the pattern of movement for different conditions and incubation times (Figure S2; Tables S1–S3). 

OTS 1 was positively correlated with linearity (LIN), straightness (STR), and wobble (WOB) in *M. musculus* and *M. spicilegus* (Figure S2D,F; Tables S1 and S3). Therefore, high OTS 1 values correspond to trajectories with low curvature and undulation in these species. In *M. spretus*, OTS 1 was only positively correlated with WOB and negatively with ALH and BCF (Figure S2E; Table S2). Thus, high OTS1 values correspond to sperm with a less undulatory pattern of movement (both in frequency and amplitude).

OTS 2 was characterized by a strong positive association with BCF in *M. musculus* and *M. spicilegus*, thus indicating the speed of sperm undulatory movement. On the other hand, in *M. spretus*, OTS 2 is mainly explained by LIN and STR (both with positive correlations). Hence, the lesser curvature of the trajectory is associated with higher OTS 2 values (Figure S2E; Table S2). 

**Figure 3:**
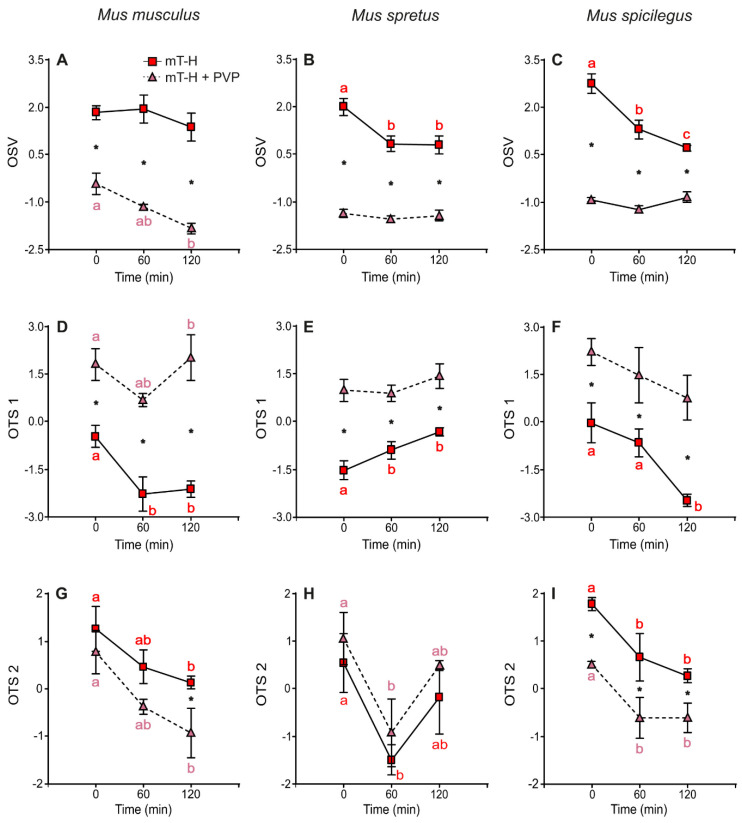
Variation in the principal components of swimming: OSV, OTS 1, and OTS 2 over time in sperm incubated in non-capacitating conditions in low viscosity (mT−H medium under air) and high viscosity (mT−H + PVP medium under air). Data are means ± SE (*M. musculus*: N = 4; *M. spretus*: N = 5; *M. spicilegus*: N = 4). (**A**–**C**): OSV. (**D**–**F**): OTS 1. (**G**–**I**): OTS 2. (**A**,**D**,**G**): *M. musculus*. (**B**,**E**,**H**): *M. spretus*. (**C**,**F**,**I**): *M. spicilegus*. Red squares and letters: non-capacitating conditions in low viscosity (mT−H). Pink triangles and letters: non-capacitating conditions in high viscosity (mT−H + PVP). Asterisks indicate significant differences between viscosity treatments for the same time point in a Fisher post-hoc test (*p* < 0.05). Different letters indicate significant differences between time points for the same viscosity treatment in a Fisher post-hoc test (*p* < 0.05).

Sperm incubated under non-capacitating conditions and low viscosity (no PVP added) showed significantly higher OSV values than sperm in high viscosity in the three species (Figure 3A–C; Table 5). In *M. musculus*, sperm velocity was stable over time in low viscosity but decreased significantly with incubation time in 2% PVP (high viscosity) (Figure 3A). Conversely, in *M. spretus* and *M. spicilegus*, OSV showed a significant decrease over time in sperm incubated in the absence of PVP (low viscosity) but remained stable in low viscosity (Figure 3B,C).

Sperm of the three species incubated under non-capacitating conditions in the absence of PVP showed significantly lower OTS 1 values than those in 2% PVP. In *M. musculus* and *M. spicilegus*, OTS 1 decreased significantly at low viscosity from 0 to 120 min. In high viscosity, there were no differences over time for *M. musculus* but there were in *M. spicilegus* (Figure 3D,F; Table 5). In *M. spretus*, OTS 1 increased during incubation in low viscosity but remained stable over time in high viscosity (Figure 3E; Table 5).

OTS 2 decreased over time and was significantly higher in sperm incubated in low viscosity in *M. musculus* and *M. spicilegus* when compared with incubation in 2% PVP (Figure 3G,I; Table 5). On the other hand, *M. spretus* sperm did not show significant differences between low or high viscosity and had a less clear pattern over time, decreasing by 60 min of incubation (Figure 3H; Table 5). 

**Figure 4:**
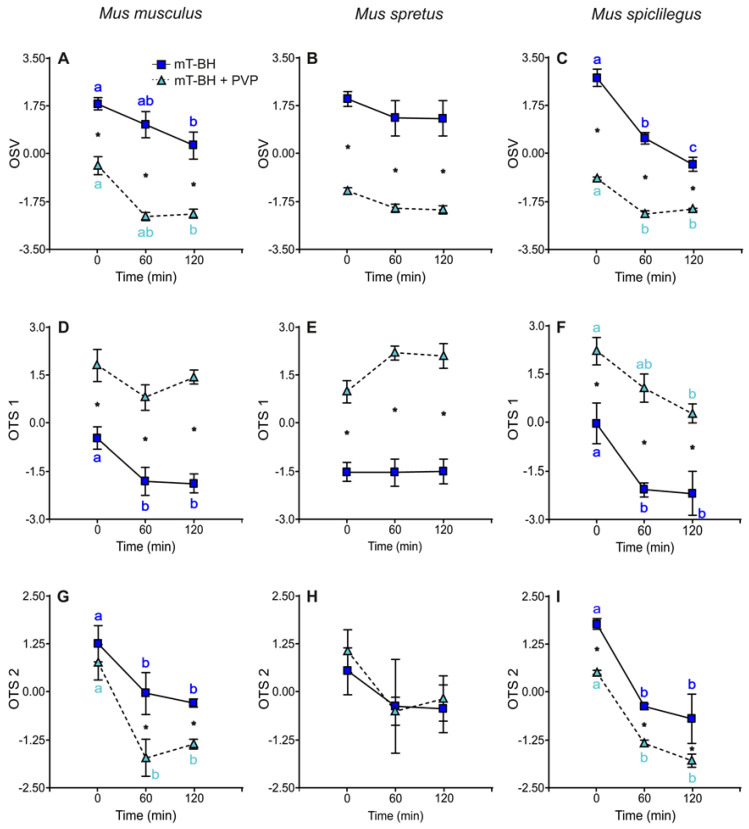
Variation in the principal components of swimming: OSV, OTS 1, and OTS 2 over time in sperm incubated in capacitating conditions in low viscosity (mT−BH medium under 5% CO_2_/air) and high viscosity (mT−BH + PVP medium under 5% CO_2_/air). Data are means ± SE (*M. musculus*: N = 4; *M. spretus*: N = 5; *M. spicilegus*: N = 4). (**A**–**C**): OSV1. (**D**–**F**): OTS 1. (**G**–**I**): OTS 2. (**A**,**D**,**G**): *M. musculus*. (**B**,**E**,**H**): *M. spretus*. (**C**,**F**,**I**): *M. spicilegus*. Dark blue squares and letters: capacitating conditions in low viscosity (mT−BH). Light blue triangles and letters: capacitating conditions in high viscosity (mT−BH + PVP). Asterisks indicate significant differences between viscosity treatments for the same time point in a Fisher post-hoc test (*p* < 0.05). Different letters indicate significant differences between time points for the same viscosity treatment in a Fisher post-hoc test (*p* < 0.05).

When incubated under capacitating conditions, sperm cells exhibited results that were similar to those observed in non-capacitating ones (see above). OSV was significantly higher in sperm incubated in low viscosity (no PVP) than in high viscosity (2% PVP) in the three species (Figure 4A–C; Table 5). In *M. musculus* and *M. spicilegus*, OSV decreased significantly in low viscosity during the incubation period but only experienced a decline from 0 min to 60 min of incubation in the high viscosity treatment (Figure 4A,C). In *M. spretus*, OSV values remained stable in sperm incubated in the absence or presence of PVP (Figure 4B). 

With regards to trajectories of sperm incubated under capacitating conditions, results showed that sperm in low viscosity exhibited values of OTS 1 that were lower than those in sperm incubated in 2% PVP in the three species (Figure 4D,F; Table 5). OTS 1 showed a significant decrease from 0 to 60 min in sperm incubated in low viscosity in *M. musculus* and *M. spicilegus* (Figure 4D,F; Table 5). Sperm incubated in high viscosity showed a linear decrease in OTS 1 throughout the incubation period in *M. spicilegus* but remained stable in *M. musculus*. In *M. spretus*, OTS 1 remained mostly stable throughout incubation time in both viscosity treatments, showing a non-significant increase at 60 min in sperm incubated in 2% PVP (Figure 4E; Table 5). OTS 2 decayed significantly with time in *M. musculus* and *M. spicilegus* but showed only a non-significant decrease in *M. spretus* in both viscosity conditions (Figure 4G,I; Table 5). This parameter was higher at all times in low viscosity conditions in *M. musculus* and *M. spicilegus* but showed no differences regarding viscosity in *M. spretus*.

#### 2.3.2. Effect of High Viscosity on ATP Content at Different Times of Incubation

**Figure 5:**
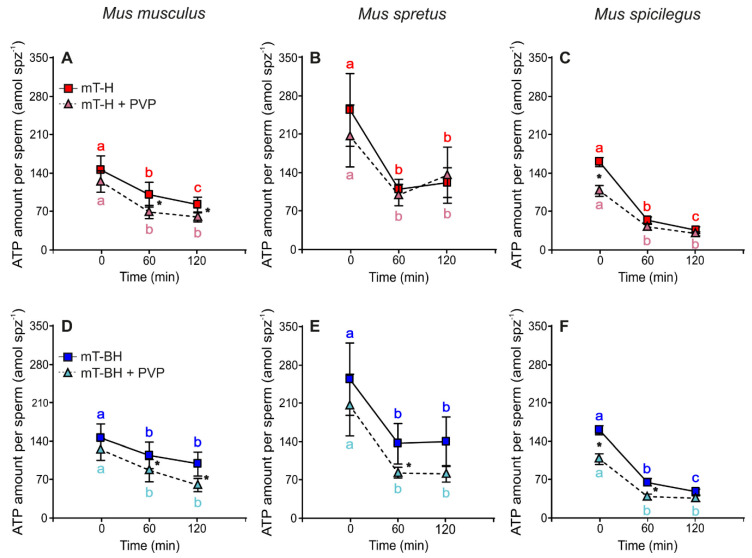
Variation in the amount of ATP per sperm over time. Data are means ± SE (*M. musculus*: N = 4; *M. spretus*: N = 5; *M. spicilegus*: N = 4). (**A**–**C**): Amount of ATP per sperm incubated in non-capacitating conditions in low viscosity (mT−H under air) and high viscosity (mT−H + PVP under air). Red squares and letters: non-capacitating conditions in low viscosity (mT−H), and pink triangles and letters: non-capacitating conditions in high viscosity (mT−H + PVP). (**D**–**F**): Amount of ATP per sperm in capacitating conditions in low viscosity (mT−BH under 5% CO_2_/air) and high viscosity (mT−BH + PVP under 5% CO_2_/air). Dark blue squares and letters: capacitating conditions in low viscosity (mT−BH), and light blue triangles and letters: capacitating conditions in high viscosity (mT-BH + PVP). (**A**,**D**): *M. musculus*. (**B**,**E**): *M. spretus*. (**C**,**F**): *M. spicilegus*. Asterisks indicate significant differences between viscosity treatments for the same time point in a Fisher post-hoc test (*p* < 0.05). Different letters indicate significant differences between time points for the same viscosity treatment in a Fisher post-hoc test (*p* < 0.05).

In non-capacitating conditions (mT-H medium), the amount of ATP decreased over time in sperm from the three species incubated in the absence or presence of 2% PVP (Figure 5A–C; Table 6). Significant differences were observed between low and high viscosity in *M. musculus* and *M. spicilegus*, with lower values of ATP in high viscosity (Figure 5A,C; Table 6). On the other hand, there were no differences in ATP values of *M. spretus* sperm incubated at various times in low or high viscosity (Figure 5B; Table 6).

Under capacitating conditions, the amount of ATP decreased significantly over time (mainly from 0 to 60 min of incubation) in the absence or presence of 2% PVP. In the three species, sperm incubated in low viscosity showed higher values of ATP amount than sperm incubated in high viscosity, although this difference was not observed at all incubation times (Figure 5D–F; Table 6). In *M. musculus*, differences were observed at and after 60 min of incubation because the amount of ATP decreased more quickly in sperm in high viscosity (Figure 5D). In *M. spretus*, significant differences were found at 60 min (Figure 5E), whereas in *M. spicilegus*, significant differences were found at 0 and 60 min (Figure 5F).

### 2.4. Comparison between Sperm Incubated in Non-Capacitating or Capacitating Conditions in High Viscosity

Results of sperm incubated in 2% PVP were compared between non-capacitating and capacitating conditions (Figure S3). In the three species, higher velocities (higher OSV values) were found in sperm in non-capacitating conditions (Table 7). In *M. musculus*, OSV decreased over time in both non-capacitating and capacitation conditions, with sperm in non-capacitating conditions declining gradually over time, whereas in capacitating conditions, the decrease was sharp, from 0 to 60 min (Figure S3A). In *M. spretus* and *M. spicilegus*, OSV only decreased in capacitation conditions between 0 and 60 min of incubation (Figure S3B,C).

In high viscosity, OTS 1 in *M. musculus* and *M. spicilegus* showed no significant differences between non-capacitating and capacitating conditions (Figure S3D,F; Table 7). *M. spretus* did exhibit significant differences between these conditions at 60 min. Whereas OTS 1 values in non-capacitating conditions remained stable, under capacitation conditions, OTS 1 showed a slight (non-significant) increase in the first 60 min of incubation (Figure S3E; Table 7). OTS 2 showed significant differences between conditions in *M. musculus* and *M. spicilegus*. Sperm incubated in capacitating conditions showed significantly higher values than sperm incubated in non-capacitating conditions (Figure S3G,I; Table 7). In *M. spretus,* no differences were found between non-capacitating and capacitating conditions (Figure S3H; Table 7). The amount of ATP decreased mainly from 0 to 60 min in the three species, but differences were not observed between non-capacitating and capacitating conditions at high viscosity (Figure S4A–C; Table 7).

## 3. Discussion

The results of this study showed that increases in viscosity in capacitating media led to different responses in three mouse species regarding sperm kinematics and ATP sperm content. While one species (*Mus musculus*) seemed to be unaffected by higher viscosities, the other two species examined (*M. spretus*, *M. spicilegus*) revealed a concentration-dependent decrease in sperm velocity and alterations in trajectory parameters. In parallel, similar differences were observed in ATP content, suggesting a relationship between energy levels and sperm movement in connection with increased viscosity. In incubations for up to 120 min, higher viscosity led to lower values of overall velocity and less linear and progressive trajectories. Although ATP levels descended during incubations for up to 120 min in capacitating conditions, there was a more pronounced descent in high viscosity than in lower viscosity.

Within the female reproductive tract, viscosity is not homogeneous. Therefore, sperm swim in a medium that may vary between 80 and 2.5 cP in the uterus and oviductal isthmus, respectively [15]. This may affect sperm kinematic parameters and overall movement patterns and, in turn, may impact energy production and usage. In this study, we modified media by the addition of polyvinylpyrrolidone (PVP) in an attempt to understand sperm behavior from three different species in response to changes in viscosity. Consistent with the hypothesis that high-viscosity media present higher physical resistance to sperm motility [47], we observed that sperm swam more slowly and with more linear trajectories in high-viscosity conditions. This agrees in general terms with previous studies in mouse [9,15,19], hamster [8], human [15], and bull sperm [47], which show the different motility characteristics of sperm in high viscosity. Underscoring the importance of viscosity in relation to capacitation and hyperactivation, a study on sperm from CatSper2^−/−^ males, which are unable to hyperactivate, revealed that they could not swim in a more viscoelastic medium [48]. 

Despite an overall tendency for altered motion in higher viscosity in many studies, some differences in our work in relation to earlier studies are worth noting. For example, an almost complete reduction of sperm velocity was observed immediately (20 s) after pre-capacitated mouse sperm were suspended in high PVP solutions, but no effects were seen on straightness or linearity [19]. In our hands, experiments carried out in lower PVP concentrations resulted in changes in sperm kinetics during the period of capacitation that could be monitored in more detail for longer, and effects on trajectories were noted. 

Analyses of the effects of viscosity (or viscoelasticity) have been carried out using several compounds. Thus, conditions and results may not be directly comparable. For instance, the use of PVP or methylcellulose results in solutions with different properties. With PVP, viscosities are constant as a function of shear. Therefore, media with PVP are representative of Newtonian fluids, with increases in PVP leading to increases in viscosity [47,49]. With methylcellulose, viscosity decreases with an increase in shear, resulting in a non-Newtonian fluid [47]. In non-Newtonian fluids, the viscosity can be altered by the application of force. With polyacrylamide, the resulting fluid is non-Newtonian, with additional viscoelastic properties [8,49]. Comparing sperm swimming parameters between non-hyperactivated and hyperactivated sperm, in a high-viscosity non-Newtonian fluid, hyperactivated bull sperm moves in a zigzag pattern with regularity, different from the movement observed in a diluted solution [49]. At similar viscosity, the VSL and flagellar beat frequency of bull spermatozoa are higher in non-Newtonian fluids, leading to a more linear motility type [47] compared with Newtonian fluids (in which, at higher viscosities, there is less sperm motility). Accordingly, it has been found that in viscoelastic conditions (non-Newtonian), hyperactive mouse spermatozoa linearize their trajectories, whereas non-hyperactive spermatozoa behave in the opposite way [9].

Importantly, in our study, the incubation of mouse sperm under capacitating conditions, in a medium containing bicarbonate, calcium, and bovine serum albumin and under 5% CO_2_/air, and with the addition of PVP to increase viscosity, did not alter the time-course or the proportion of cells experiencing changes compatible with capacitation. The use of a Hoechst 33258 stain to identify live cells, followed by staining with chlortetracycline [34,44,45,50,51,52], revealed no differences between sperm incubated in the absence or the presence of PVP with regards to the proportion of spermatozoa exhibiting a B staining pattern, indicative of capacitation. This was observed in each of the three mouse species examined in this study. This seems to be similar to a lack of effect of higher viscosity on the capacitation of bull spermatozoa in vitro [53]. It follows that the differences in sperm motion parameters or ATP levels at high viscosity observed here are most likely not due to an effect on capacitation per se. Rather, the most likely explanation is that high viscosity affects sperm kinetics and energy production. The impact on ATP levels could be linked to a higher energetic demand for swimming in higher viscosity, but this should be examined further.

Sperm motility has a substantial energy demand, with the amount of intracellular ATP strongly related to sperm kinetics and the intensity of flagellar beating [21,22,23,24,25,33,54]. The spermatozoa of the mouse species examined in this study can maintain high concentrations of intracellular ATP and swimming velocity over time, suggesting a positive balance between ATP production and consumption, although differences between species exist [25,33]. *M. musculus* sperm ATP levels did not appear to be affected by high viscosity. In the other two species, *M. spretus* and *M. spicilegus*, differences in the amount of ATP between low and high viscosity in both non-capacitating and capacitating conditions were found. Interestingly, our previous studies revealed that sperm from *M. musculus* experienced a similar decrease in ATP levels over time of incubation in non-capacitating and capacitating cells. On the other hand, a descent in ATP with time was also seen in the other two species; however, in addition, differences between non-capacitating and capacitating sperm ATP levels were recorded [34,39]. Recent work on the sperm of laboratory mouse strains found that these cells increase the uptake of glucose and undergo changes in their metabolic rates when exposed to capacitating conditions [35,39,55]. These changes can be interpreted as metabolic responses to sustain ATP levels in the face of increased ATP demands caused by capacitation.

The question that now arises is whether slower motility due to higher viscosity reflects on ATP levels. That is, does ATP decrease because of the increased energy consumption imposed by the higher flagellar beating force required to overcome a more viscous fluid? The results showed that spermatozoa in a more viscous medium exhibited an alteration in kinetic parameters: sperm became slower, both progressively and laterally, in two species (but not in the other). Concomitantly, there was a decrease in ATP levels in species exhibiting reduced kinetics, so there seems to be an association between motion and ATP in more viscous conditions. A relationship between ATP levels and sperm swimming traits was previously seen in experimental and comparative studies [24,25]. The next question to be considered is whether sperm would produce less ATP in high viscosity or would consume more ATP. Studies in bull sperm using extracellular flux analyses revealed that there were no apparent differences in extracellular acidification rates (ECAR, indicative of glycolysis) or oxygen consumption rates (OCR, indicative of oxidative phosphorylation) in cells incubated in low or high viscosity [56], which suggests that ATP production may not be affected. Thus, the consumption of ATP may vary with the increased energetic demand resulting from movement in high viscosity. Future studies should examine this possibility in more detail.

Sperm motility, bioenergetics, and sperm behavior at varying viscosities could relate to the selective forces under which these species evolved. Postcopulatory sexual selection in the form of sperm competition is known to promote adaptations on several sperm parameters and the male’s fertilizing capacity [57,58]. Sperm competition occurs when the ejaculates from different males compete to fertilize a set of ova as a consequence of female promiscuity [59]. The three species examined in the present study are known to differ in their intensities of sperm competition [60]. Thus, differences observed in sperm kinetics and sperm ATP content of non-capacitating and capacitating sperm in media with varying viscosities may be the result of responses to different levels of sperm competition. The species used in this study represent a closely related monophyletic group, with an estimated divergence time of 1.7 million years [61,62]. Despite their phylogenetic proximity, these species show significant differences in sperm traits such as numbers [63], morphology [64], percentage of motile cells [46], swimming velocity [25,33,46], and capacitation regime [34,60]. Moreover, there are also substantial differences between these species regarding sperm ATP levels [24,25], ATP production pathways [34,65], and ATP consumption rates [33]. Such divergences have been interpreted as results of the interspecific differences in the levels of sperm competition experienced by the males [60].

Lastly, it is worth speculating about possible applications of media with increased viscosity to capacitating or non-capacitating domestic animal and human sperm assessments. For example, incubations of spermatozoa in glass capillary tubes containing methylcellulose and the assessment of migration distance have been proposed [66]. There was a highly significant correlation between the number of spermatozoa at all migration distances in methylcellulose and semen parameters (motility, concentration, normality) and effective discrimination between normal and abnormal human sperm samples [66]. It is also possible to select (non-capacitating) bull or human sperm in a microfluidic device containing a medium with high viscosity [67,68]. A selection of sperm subpopulations with a 130–275% increase in sperm viability was obtained, depending on the compound used to enhance viscosity. In methylcellulose, sperm had slower velocities and swam straighter [67]. In human sperm, sperm selected in a microfluidic device with high viscosity had, upon recovery, a higher proportion of motile sperm [68]. In pig in vitro fertilization, the incubation of boar sperm in a viscous medium improved quality and penetration rates [69].

In conclusion, we did not find differences in the ability of sperm to undergo changes compatible with capacitation between low or high viscosity. That is, high viscosity did not appear to affect the onset of capacitation, but it did affect sperm motion and concomitant ATP levels. It remains to be established whether lower ATP content is the result of a higher effort in motility or if it is due to other causes. In addition, future studies may wish to characterize the effect of different viscous or viscoelastic conditions on sperm bioenergetics.

## 4. Materials and Methods

### 4.1. Animals and Sperm Collection 

Adult males (4–6 months) from three mouse species, *Mus musculus* (N = 8), *M. spretus* (N = 12), and *M. spicilegus* (N = 11), were used in this study. The individuals were purchased from the Institut des Sciences de l’Evolution, CNRS-Université Montpellier 2, France. Animals were maintained under standard conditions (14 h light–10 h darkness at 22–24 °C) with food and water ad libitum. All animal handling was done following Spanish Animal Protection Regulation RD53/2013, which conforms to European Union Regulation 2010/63, and had the approval of CSIC’s ethics committee and the Comunidad de Madrid (28079-47-A).

Males were sacrificed by cervical dislocation and dissected immediately. Both caudae epididymides were removed and placed in a Petri dish containing a fixed volume of culture medium for each species at 37 °C [34]. The culture medium was a Hepes-buffered modified Tyrode’s medium (mT-H), the composition of which was 131.89 mM NaCl, 2.68 mM KCl, 0.49 mM MgCl_2_·6 H_2_O, 0.36 mM NaH_2_PO_4_·2H_2_O, 5.56 mM glucose, 20 mM lactate, 0.5 mM pyruvate, 20.00 mM Hepes, 5 µg mL^−1^ phenol red, 50 µg mL^−1^ kanamycin, and 4 mg mL^−1^ fatty acid-free bovine serum albumin (BSA) [70]. The pH of the medium was adjusted to 7.4, and it had an osmolality of 295 mOsm kg^−1^. After 10 min incubation under air (swim-out), the epididymides were removed. Sperm concentration was estimated by using a Neubauer chamber, and the suspension was adjusted to 40 × 10^6^ sperm mL^−1^ by adding mT-H. Then, the sperm suspension was separated into four aliquots, and an equivalent volume of the appropriate medium (according to each treatment; see below) was added to each aliquot to obtain a working concentration of ~20 × 10^6^ sperm mL^−1^. To incubate sperm under non-capacitating conditions, the mT-H medium described above was used. To incubate sperm under capacitating conditions, a modification of this medium was employed. The capacitating medium consisted of a variation of the medium above, with final concentrations of 15 mM NaHCO_3_ and 116.89 mM NaCl (the latter adjusted to maintain the same osmolality) [70]. Spermatozoa were incubated under air (with mT-H) or 5%CO_2_/air (with mT-BH).

### 4.2. Experimental Design: Effects of Low and High Viscosities

Experiments were carried out to examine the effects of viscosity on ATP levels and sperm kinematics. Two series of experiments were carried out to assess: (a) the effects of incubation at different viscosities or (b) the effects of low versus high viscosity over time. 

In the first series of experiments, sperm from *M. musculus* (4 individuals), *M. spretus* (7 individuals), and *M. spicilegus* (7 individuals) were incubated at 37 °C in mT-H medium under air (non-capacitating conditions) or mT-BH medium under 5% CO_2_/air (capacitating conditions), with 0%, 1%, 2%, or 4% polyvinylpyrrolidone (PVP) (M_w_ 360,000, Sigma, Madrid, Spain). Sperm kinetic parameters were assessed in spermatozoa after a 30 min incubation. ATP levels were examined in sperm incubated for 0, 15, or 30 min. 

In the second series of experiments, the effects of low or high viscosity over time were analyzed. Sperm from *M. musculus* (4 individuals), *M. spretus* (5 individuals), and *M. spicilegus* (4 individuals) were incubated at 37 °C in non-capacitating conditions (mT-H under air) or capacitating conditions (mT-BH medium under 5% CO_2_/air) with 0% or 2% PVP. Samples were collected for kinetic analyses and ATP level assessments at 0, 60, and 120 min. Higher PVP concentrations were not used in these experiments because preliminary experiments revealed that they render sperm motionless after a short period. 

### 4.3. Sperm Viability and Motility 

Sperm viability was assessed in sperm smears stained with eosin–nigrosine and Giemsa. A volume of 5 µL of sperm suspension was mixed with 10 µL of eosin–nigrosin solution on a glass slide preheated at 37 °C; after 30 s, the mix was smeared and left to dry, and subsequently, smears were stained with Giemsa solution and were mounted with DePeX [63]. Smears were examined in a microscope with an oil-immersion 100× objective under a bright field. A total of 100 spermatozoa were assessed. Viable sperm with intact acrosomes showed a clear post-acrosomal region (not stained with eosin) and a purple-stained acrosome (stained with Giemsa).

Sperm motility was evaluated on a 10 µL aliquot of sperm suspension. The aliquot was placed on a glass slide preheated at 37 °C and was covered with a 22 × 22 mm coverslip. The preparation was observed with a phase-contrast optical microscope bearing a 10× objective. The percentage of motile sperm was observed by two experienced observers subjectively and averaged. The estimates were averaged to the nearest 5% value.

### 4.4. Sperm Capacitation

Sperm capacitation was assessed by combined fluorescent staining using chlortetracycline (CTC) and Hoechst 33258 bisbenzamide. Samples were stained as previously described [52,71]. Briefly, Hoechst 33258 was added to the sperm sample (6 µg/mL final concentration), and the sample was incubated for 1 min at 37 °C in the dark. Subsequently, the sample was centrifuged for 3 min at 100× *g*, the supernatant was discarded, and the pellet was resuspended with incubation medium (mT-H or mT-BH, according to treatment) and fixed with a solution of 2.5% glutaraldehyde-sodium phosphate. The stained sample was stored in the dark at 4 °C (a maximum of 12 h) until evaluation. At the time of evaluation, CTC solution (concentration: 250 µM) was added, and the sample was incubated in the dark for 3 min at room temperature. The stained preparations were observed using a 100× oil-immersion objective with fluorescence and phase-contrast optical microscopy simultaneously. Pre-fixation viability of the spermatozoa was assessed using a Nikon UV-2A 330 nm filter and fluorescence emission via a DM 400 dichroic mirror. Only cells that did not stain with Hoechst 33258 were considered viable and assessed for capacitation. Capacitated status was evaluated by observation of the CTC staining patterns of 100 viable cells from each sample using a Nikon BV-2A 405 nm filter and fluorescence emission with a DM 455 dichroic mirror. The presence or absence of the acrosome was evaluated with phase contrast. The patterns observed were: (a) CTC pattern F (non-capacitated sperm): sperm head uniformly stained with CTC and intact acrosome; (b) CTC pattern B (capacitated sperm): post-acrosomal region of the sperm head not stained with CTC and intact acrosome; (c) CTC pattern AR (without acrosome): sperm head not stained with CTC (or slightly stained) [34,71].

### 4.5. Sperm Swimming Parameters

Sperm swimming parameters were evaluated in a diluted aliquot of sperm suspension (4–6 × 10^6^ sperm mL^−1^) for each treatment. A 6 µL aliquot was placed in a pre-warmed 20 µm chamber (Leja, Nieuw-Vennep, The Netherlands). Sperm were filmed using a videomicroscopy system consisting of a phase-contrast microscope (Nikon CI, Tokyo, Japan) with a 4× pseudo-negative phase objective and a digital video camera (Basler A312fc, Vision Technologies, Gien Burnie, MD, USA). Videos were captured and analyzed using the software Sperm Class Analyzer (SCA, v. 6.0., Microptic, Barcelona, Spain). Six randomly selected fields were recorded at 75 frames s^−1^, with a minimum of 150 spermatozoa per sample. The software parameters were: minimum particle size, 50 µm; maximum particle size, 320 µm; contrast, 600; brightness, 60; and connectivity, 20. The following kinetic parameters were measured: curvilinear velocity (µm s^−1^) (VCL), straight-line velocity (µm s^−1^) (VSL), average path velocity (VAP) (µm s^−1^), linearity (%) (LIN = VSL/VCL), straightness (%) (STR = VSL/VAP), wobble coefficient (%) (WOB =VAP/VCL), amplitude of lateral head displacement (µm) (ALH), and beat cross frequency (Hz) (BCF). The trajectories where VAP was lower than 20 µm s^−1^ were eliminated so that drifting particles and immotile sperm were eliminated.

### 4.6. Sperm ATP Content

Sperm ATP content was measured using a luciferase-based ATP bioluminescence assay kit (Roche, ATP Bioluminescence Assay Kit HS II, Roche Farma, Madrid, Spain) as previously described [24,25,65]. A 10 µL aliquot from each treatment was taken and added to 90 µL medium and 100 µL Cell Lysis Reagent. Subsequently, the sample was homogenized by vortex agitation, incubated for 5 min at room temperature, and centrifuged at 12,000× *g* for 2 min. A total of 50 µL of supernatant was recovered and added to 450 µL mT-H medium and immediately frozen in liquid N_2_ to prevent ATP degradation. The diluted supernatant (samples) was conserved at −80 °C until evaluation.

At the time of evaluation of ATP content, a standard curve was generated using known concentrations of ATP diluted in Dilution Buffer with mT-H and Cell Lysis Reagent in a proportion equivalent to that of the samples. Samples and standards were measured in triplicate in 96-well plates using a luminometer (BioTek Sinergy HT, Winooski, VT, USA). Each well was loaded with 50 µL of the sample, and 50 µL of luciferase was added via auto-injection. After a 1 s delay, light emission was measured over a 10 s integration period. The ATP content of each sample (amol sperm^−1^) was calculated by converting the average of the bioluminescence values of three wells and using the conversion rule estimated from the standard curve. 

### 4.7. Statistical Analysis

Results are shown as mean ± standard error. Statistical analyses were performed with SPSS Statistics 23.0 statistic software (IBM, Armonk, NY, USA), InfoStat 2017 statistic software (FCA, Universidad Nacional de Córdoba, Argentina), or GraphPad Prism 9 software (Dotmatics, CA, USA). Repeated measures analyses (ANOVAs) were performed using treatment (mT-H, mT-H + PVP, mT-BH, and mT-BH + PVP) and time (0, 60, and 120 min) as fixed variables and the individual as a random variable for each species. In addition, Fisher LSD post-hoc tests were used to compare each treatment and time in pairs. The level of statistical significance was 0.05 (*α* = 0.05). 

For dose–response studies, a two-way ANOVA with Tukey’s post-hoc test was performed to evaluate significant differences (*p* < 0.05) in ATP levels at different times (0, 15, and 30 min) and with 0%, 1%, 2%, or 4% PVP. A one-way ANOVA was performed for the assessment of differences in sperm kinematic parameters with different PVP concentrations at 30 min. Significant differences (*p* < 0.05–0.0001) are given. 

## Figures and Tables

**Table 1:** Effect of various concentrations of PVP on the kinetics of spermatozoa from *Mus musculus* incubated under non-capacitating conditions (mT-H medium under air) or capacitating conditions (mT-BH medium under 5%CO_2_/air) for 30 min. VCL: curvilinear velocity; VSL: straight-line velocity; VAP: average path velocity; LIN: linearity; STR: straightness; WOB: wobble coefficient; ALH: amplitude of lateral head; BCF: beat cross frequency. Means ± SE (N = 4) are reported. Letters indicate different levels of significance between different PVP concentrations and control (0% PVP) in Tukey’s post-hoc test (^a^: *p* < 0.05).

Swimming Parameter	Non-Capacitating Conditions				Capacitating Conditions			
	% PVP				% PVP			
	0	1	2	4	0	1	2	4
VCL	149.2 ± 9.8	139.2 ± 9.5	136.1 ± 9.1	125.1 ± 7.9 ^a^	144.4 ± 14.0	123.1 ± 40.0	149.5 ± 19.2	120.8 ± 2.0
VSL	42.4 ± 8.6	38.3 ± 3.6	40.9 ± 2.8	34.8 ± 1.8	40.8 ± 6.0	35.7 ± 10.1	45.0 ± 6.1	34.0 ± 1.7
VAP	69.6 ± 9.4	63.5 ± 4.3	65.9 ± 3.9	60.4 ± 0.2	66.6 ± 7.9	58.4 ± 19.3	71.7 ± 7.6	57.4 ± 0.3
LIN	28.9 ± 2.7	29.0 ± 2.2	31.4 ± 2.7	30.8 ± 1.3	28.2 ± 3.1	28.1 ± 4.0	30.9 ± 3.0	31.2 ± 0.3
STR	55.9 ± 4.2	56.4 ± 2.0	57.1 ± 3.0	55.1 ± 2.4	56.4 ± 2.4	52.0 ± 7.7	57.1 ± 2.5	56.4 ± 3.0
WOB	48.5 ± 2.6	48.0 ± 1.9	51.7 ± 1.5	52.4 ± 2.8	47.1 ± 3.3	47.4 ± 6.9	50.9 ± 2.4	51.7 ± 1.3
ALH	3.3 ± 0.2	3.2 ± 0.1	3.2 ± 0.1	3.1 ± 0.2	3.3 ± 0.2	2.8 ± 1.0	3.3 ± 0.3	3.1 ± 0.2
BCF	24.7 ± 1.6	23.7 ± 1.2	22.9 ± 0.6	21.4 ± 3.0	23.6 ± 2.7	23.0 ± 0.9	22.1 ± 0.9	20.7 ± 0.4

**Table 2:** Effect of various concentrations of PVP on the kinetics of spermatozoa from *Mus spretus* incubated under non-capacitating conditions (mT-H medium under air) or capacitating conditions (mT-BH medium under 5%CO_2_/air) for 30 min. VCL: curvilinear velocity; VSL: straight-line velocity; VAP: average path velocity; LIN: linearity; STR: straightness; WOB: wobble coefficient; ALH: amplitude of lateral head; BCF: beat cross frequency. Means ± SE (N = 7) are reported. Letters indicate different levels of significance between different PVP concentrations and control (0% PVP) in Tukey’s post-hoc test (^a^: *p* < 0.05; ^b^: *p* < 0.01; ^c^: *p* < 0.001; ^d^: *p* < 0.0001).

Swimming Parameter	Non-Capacitating Conditions				Capacitating Conditions			
	% PVP				% PVP			
	0	1	2	4	0	1	2	4
VCL	244.0 ± 36.8	193.6 ± 25.6 ^b^	159.4 ± 23.6 ^d^	139.0 ± 8.1 ^d^	247.8 ± 45.8	168.8 ± 20.9 ^c^	141.9 ± 29.6 ^d^	131.3 ± 18.4 ^d^
VSL	76.6 ± 19.4	61.3 ± 13.1	46.3 ± 9.1 ^b^	39.6 ± 5.0 ^c^	82.2 ± 22.6	44.8 ± 10.9 ^c^	38.2 ± 12.4 ^d^	35.1 ± 7.2 ^d^
VAP	108.7 ± 18.5	90.2 ± 15.9	74.5 ± 12.8 ^b^	66.4 ± 7.8 ^d^	116.2 ± 24.6	74.6 ± 13.9 ^c^	63.1 ± 16.7 ^d^	59.9 ± 11.6 ^d^
LIN	30.3 ± 3.7	30.3 ± 3.6	29.0 ± 2.1	30.0 ± 2.4	29.6 ± 3.1	26.0 ± 3.9	26.1 ± 4.3	27.4 ± 2.6
STR	61.6 ± 6.2	61.5 ± 4.1	57.6 ± 2.8	56.7 ± 2.4	61.1 ± 4.0	55.3 ± 3.1 ^a^	56.0 ± 3.4	55.4 ± 3.7 ^a^
WOB	46.1 ± 3.3	46.8 ± 4.0	47.8 ± 2.3	49.8 ± 3.3	46.2 ± 3.5	44.7 ± 4.6	44.4 ± 5.9	46.8 ± 4.6
ALH	5.0 ± 0.6	4.5 ± 0.6	4.0 ± 0.4 ^b^	3.6 ± 0.3 ^c^	5.2 ± 1.3	4.1 ± 0.3	3.6 ± 0.6 ^b^	3.4 ± 0.2 ^c^
BCF	25.8 ± 3.8	23.6 ± 1.7	23.3 ± 0.8	22.5 ± 0.8	23.9 ± 3.4	22.6 ± 2.8	22.3 ± 2.2	21.5 ± 2.6

**Table 3:** Effect of various concentrations of PVP on the kinetics of spermatozoa from *Mus spicilegus* incubated under non-capacitating conditions (mT-H medium under air) or capacitating conditions (mT-BH medium under 5%CO_2_/air) for 30 min. VCL: curvilinear velocity; VSL: straight-line velocity; VAP: average path velocity; LIN: linearity; STR: straightness; WOB: wobble coefficient; ALH: amplitude of lateral head; BCF: beat cross frequency. Means ± SE (N = 7) are reported. Letters indicate different levels of significance between different PVP concentrations and control (0% PVP) in Tukey’s post-hoc test (^a^: *p* < 0.05; ^b^: *p* < 0.01; ^c^: *p* < 0.001; ^d^: *p* < 0.0001).

Swimming Parameter	Non-Capacitating Conditions				Capacitating Conditions			
	% PVP				% PVP			
	0	1	2	4	0	1	2	4
VCL	222.1 ± 45.4	191.5 ± 32.0	140.7 ± 19.2 ^b^	115.5 ± 21.2 ^d^	171.3 ± 30.3	123.7 ± 21.9 ^b^	128.5 ± 9.6 ^a^	105.1 ± 18.5 ^c^
VSL	88.2 ± 22.3	62.0 ± 14.8 ^a^	53.5 ± 10.6 ^b^	36.7 ± 7.6 ^d^	57.6 ± 19.4	43.7 ± 12.3	41.8 ± 3.0	29.3 ± 3.8 ^b^
VAP	94.9 ± 36.9	91.3 ± 19.1	79.9 ± 11.2	63.6 ± 16.9	88.7 ± 25.9	84.4 ± 18.7	66.2 ± 4.1	50.2 ± 6.4 ^b^
LIN	38.2 ± 5.2	36.4 ± 3.9	36.6 ± 2.0	33.0 ± 3.7	31.8 ± 6.9	32.8 ± 5.0	31.7 ± 6.0	30.1 ± 3.9
STR	67.7 ± 3.8	66.5 ± 5.0	63.2 ± 2.9	59.0 ± 3.8 ^b^	59.0 ± 6.1	58.7 ± 6.7	57.5 ± 2.9	55.9 ± 2.6
WOB	53.1 ± 5.5	51.9 ± 2.6	54.9 ± 3.6	52.7 ± 3.9	51.0 ± 9.0	53.6 ± 7.8	52.1 ± 9.5	51.5 ± 6.0
ALH	4.4 ± 1.3	4.0 ± 0.7	3.6 ± 0.4	3.3 ± 0.6	4.0 ± 0.7	3.4 ± 0.6	3.0 ± 0.4 ^a^	3.0 ± 0.3 ^b^
BCF	26.0 ± 1.8	24.4 ± 1.7	23.7 ± 1.6	21.0 ± 1.4 ^c^	20.8 ± 2.9	21.0 ± 4.1	20.2 ± 2.5	18.8 ± 2.2

**Table 4:** Effect of various concentrations of PVP on ATP levels in spermatozoa from three mouse species incubated for different lengths of time under non-capacitating conditions (mT-H medium under air) or capacitating conditions (mT-BH medium under 5%CO_2_/air). ATP values correspond to ATP amount per sperm (amol spz^−1^). Means ± SE (*M. musculus*: N = 4; *M. spretus*: N = 7; *M. spicilegus*: N = 7) are reported. Letters in the same row indicate significant differences between different PVP concentrations and control (0% PVP) in Tukey’s post-hoc test (^a^: *p* < 0.05). Asterisks in the same column indicate significant differences between times in comparison to time 0 for the same PVP concentration in Tukey’s post-hoc test (*p* < 0.05).

Time (min)	Non-Capacitating				Capacitating			
	% PVP				% PVP			
	0	1	2	4	0	1	2	4
*M. musculus*								
0	87.7 ± 40.2	68.9 ± 27.5	87.6 ± 24.8	68.0 ± 29.5	82.1 ± 31.9	85.8 ± 29.7	78.9 ± 24.2	90.9 ± 34.6
15	79.9 ± 37.9	69.7 ± 34.1	65.1 ± 40.8	70.2 ± 30.9	69.2 ± 29.4 *	75.5 ± 31.7 *	76.6 ± 33.1	84.1 ± 56.8
30	70.4 ± 34.0	58.7 ± 29.2	96.2 ± 59.7	83.4 ± 35.2	65.7 ± 39.2 *	79.5 ± 42.0	64.4 ± 37.6	90.3 ± 67.8
*M. spretus*								
0	354.6 ± 69.9	296.6 ± 76.4	274.5 ± 55.3	257.0 ± 68.8	291.3 ± 49.6	266.2 ± 61.7	277.0 ± 53.6	170.6 ± 48.6 ^a^
15	263.9 ± 83.2 *	271.7 ± 88.6	334.8 ± 50.3	219.1 ± 36.2	248.6 ± 32.7	279.8 ± 16.3	257.5 ± 38.9	182.3 ± 61.0
30	258.9 ± 75.8 *	203.1 ± 63.6	198.2 ± 65.6	153.8 ± 48.9 *	202.6 ± 57.7 *	179.7 ± 62.4 *	151.3 ± 66.7	124.4 ± 18.1 ^a^
*M. spicilegus*								
0	99.2 ± 50.8	98.4 ± 59.0	82.2 ± 34.1	90.3 ± 52.3	89.1 ± 42.5	57.9 ± 30.8	78.9 ± 50.5	77.3 ± 62.4
15	96.9 ± 41.5	81.3 ± 44.0	62.9 ± 36.0 *	36.3 ± 27.6	73.1 ± 28.7	45.5 ± 19.3	49.9 ± 28.0	22.6 ± 16.0
30	72.5 ± 24.4	57.7 ± 28.0	47.3 ± 18.3 *	30.0 ± 22.0	61.4 ± 23.1	30.5 ± 12.1 ^a^	25.1 ± 16.8 ^a^	16.0 ± 9.3 ^a^

**Table 5:** Effect of time and incubation in two non-capacitating treatments (low and high viscosity) and two capacitating conditions (low and high viscosity) on overall sperm velocity (OSV) and overall trajectory shape (OTS 1 and OTS 2). Values of *F* and *p* are the result of repeated measures analyses (ANOVAs). Incubation conditions and time of incubation were defined as fixed factors, and the individual was defined as a random factor. Results highlighted in bold show significant differences in statistical analysis (*p* < 0.05).

	Dependent Variable	Independent Variable	*Mus* *musculus*		*Mus* *spretus*		*Mus* *spicilegus*	
			*F*	*p*	*F*	*p*	*F*	*p*
Non-capacitating conditions	OSV	Viscosity	133.52	**<0.0001**	227.53	**<0.0001**	290.01	**<0.0001**
		Time	4.91	**0.023**	6.63	**0.011**	15.53	**<0.0001**
		Interaction	1.5	0.256	4.94	0.27	16.12	**<0.0001**
	OTS 1	Viscosity	67.30	**<0.0001**	63.85	**<0.0001**	34.32	**<0.0001**
		Time	4.98	**0.019**	3.63	**0.05**	6.67	**0.009**
		Interaction	2.08	0.153	1.05	0.372	0.83	0.454
	OTS 2	Viscosity	6.56	**0.02**	1.52	0.23	19.02	**0.001**
		Time	7.16	**0.005**	6.61	**0.008**	9.21	**0.003**
		Interaction	0.29	0.753	0.01	0.992	0.26	0.775
Capacitating conditions	OSV	Viscosity	108.25	**<0.0001**	201.64	**<0.0001**	266.43	**<0.0001**
		Time	14.94	**<0.0001**	2.28	0.134	60.76	**<0.0001**
		Interaction	1.72	0.212	0.14	0.868	12.51	**<0.0001**
	OTS 1	Viscosity	82.52	**<0.0001**	228.50	**<0.0001**	46.55	**<0.0001**
		Time	5.87	**0.014**	2.42	0.121	10.13	**0.001**
		Interaction	0.84	0.453	2.43	0.121	0.51	0.608
	OTS 2	Viscosity	10.29	**0.006**	0.08	0.787	32.4	**<0.0001**
		Time	13.61	**0.001**	2.16	0.147	56.29	**<0.0001**
		Interaction	1.08	0.365	0.08	0.927	0.22	0.806

**Table 6:** Effect of time and incubation in two non-capacitating treatments (low and high viscosity) and two capacitating conditions (low and high viscosity) on ATP amount per sperm. Values of *F* and *p* are the result of repeated measures analyses (ANOVAs). Incubation conditions and time incubation were defined as fixed values, and the individual was defined as a random factor. Results highlighted in bold show significant differences in statistical analysis (*p* < 0.05).

	Dependent Variable	Independent Variable	*Mus* *musculus*		*Mus* *spretus*		*Mus* *spicilegus*	
			*F*	*p*	*F*	*p*	*F*	*p*
Non-capacitating conditions	ATP amount per sperm (amol spz^−1^)	Viscosity	11.42	**0.004**	0.77	0.395	31.27	**<0.0001**
		Time	26.75	**<0.0001**	32.46	**<0.0001**	217.90	**<0.0001**
		Interaction	0.221	0.804	1.17	0.336	11.15	**0.001**
Capacitating conditions	ATP amount per sperm (amol spz^−1^)	Viscosity	34.46	**<0.0001**	6.33	**0.021**	39.03	**<0.0001**
		Time	42.77	**<0.0001**	14.25	**<0.0001**	145.39	**<0.0001**
		Interaction	1.19	0.330	0.02	0.977	5.99	**0.012**

**Table 7:** Effect of time of incubation in non-capacitating or capacitating conditions (mT-H or mT-BH media, respectively) in high viscosity (2% PVP) on overall sperm velocity (OSV), overall trajectory shape (OTS 1 and OTS 2), and amount of ATP. Values of *F* and *p* are the results of repeated measures analyses (ANOVAs). Incubation conditions and time of incubation were defined as fixed factors, and the individual was defined as a random factor. Results highlighted in bold show significant differences in statistical analysis (*p* < 0.05).

Dependent Variable	Independent Variable	*Mus* *musculus*		*Mus* *spretus*		*Mus* *spicilegus*	
		*F*	*p*	*F*	*p*	*F*	*p*
OSV	Medium	10.83	**0.005**	26.68	**<0.0001**	66.23	**<0.0001**
	Time	40.75	**<0.0001**	22.41	**<0.0001**	25.64	**<0.0001**
	Interaction	4.97	**0.022**	8.28	**0.005**	15.59	**<0.0001**
OTS 1	Medium	0.3	0.592	5.92	**0.027**	0.38	0.545
	Time	5.87	**0.013**	3.11	0.072	3.74	**0.046**
	Interaction	0.6	0.563	1.97	0.172	0.09	0.916
OTS 2	Medium	5.15	**0.038**	0.9	0.361	10.02	**0.007**
	Time	22.68	**<0.0001**	30.87	**<0.0001**	25.19	**<0.0001**
	Interaction	2.30	0.134	2.64	0.112	2.73	0.101
ATP amount per sperm (amol spz^−1^)	Medium	1.16	0.299	0.34	0.569	0.03	0.861
	Time	44.7	**<0.0001**	13.8	**<0.0001**	87.97	**<0.0001**
	Interaction	1.03	0.380	0.36	0.701	0.26	0.775

## Data Availability

The raw data supporting the conclusions of this article will be made available by the authors without undue reservation.

## Supplementary Materials

The following supporting information can be downloaded at: https://www.mdpi.com/article/10.3390/ijms232315247/s1.

## References

[1] Austin C.R. (1952). The capacitation of the mammalian sperm. Nature.

[2] De Jonge C. (2005). Biological basis for human capacitation. Hum. Reprod. Update.

[3] De Jonge C. (2017). Biological basis for human capacitation-revisited. Hum. Reprod. Update.

[4] Nixon B., Bromfield E.G., Skinner M.K. (2018). Sperm capacitation. Encyclopedia of Reproduction.

[5] Yanagimachi R., Mastroiani L., Biggers J.D. (1981). Mechanisms of fertilization in mammals. Fertilization and Embryonic Development In Vitro.

[6] Suarez S.S., Osman R.A. (1987). Initiation of hyperactivated flagellar bending in mouse sperm within the female reproductive tract. Biol. Reprod..

[7] Goodson S.G., Zhang Z., Tsuruta J.K., Wang W., O’Brien D.A. (2011). Classification of mouse sperm motility patterns using an automated multiclass support vector machines model. Biol. Reprod..

[8] Suarez S.S., Katz D.F., Owen D.H., Andrew J.B., Powell R.L. (1991). Evidence of the funciton of hyperactivated motility in sperm. Biol. Reprod..

[9] Suarez S.S., Dai X. (1992). Hyperactivation enhances mouse sperm capacity for penetrating viscoelastic media. Biol. Reprod..

[10] Tung C.K., Lin C., Harvey B., Fiore A.G., Ardon F., Wu M., Suarez S.S. (2017). Fluid viscoelasticity promotes collective swimming of sperm. Sci. Rep..

[11] Qu Y., Chen Q., Guo S., Ma C., Lu Y., Shi J., Liu S., Zhou T., Noda T., Qian J. (2021). Cooperation-based sperm clusters mediate sperm oviduct entry and fertilization. Protein Cell.

[12] Phuyal S., Suarez S.S., Tung C.K. (2022). Biological benefits of collective swimming of sperm in a viscoelastic fluid. Front. Cell Dev. Biol..

[13] Jansen R.P. (1978). Fallopian tube isthmic mucus and ovum transport. Science.

[14] Smith D.J., Gaffney E.A., Gadelha H., Kapur N., Kirkman-Brown J.C. (2009). Bend propagation in the flagella of migrating human sperm, and its modulation by viscosity. Cell Motil. Cytoskelet..

[15] Miki K., Clapham D.E. (2013). Rheotaxis guides mammalian sperm. Curr. Biol..

[16] Armon L., Eisenbach M. (2011). Behavioral mechanism during human sperm chemotaxis: Involvement of hyperactivation. PLoS ONE.

[17] Boryshpolets S., Perez-Cerezales S., Eisenbach M. (2015). Behavioral mechanism of human sperm in thermotaxis: A role for hyperactivation. Hum. Reprod..

[18] Kantsler V., Dunkel J., Blayney M., Goldstein R.E. (2014). Rheotaxis facilitates upstream navigation of mammalian sperm cells. eLife.

[19] Perez-Cerezales S., Lopez-Cardona A.P., Gutierrez-Adan A. (2016). Progesterone effects on mouse sperm kinetics in conditions of viscosity. Reproduction.

[20] Bohnensack R., Halangk W. (1986). Control of respiration and of motility in ejaculated bull spermatozoa. Biochim. Biophys. Acta.

[21] Jeulin C., Soufir J.C. (1992). Reversible intracellular ATP changes in intact rat spermatozoa and effects on flagellar sperm movement. Cell Motil. Cytoskelet..

[22] Berlinguer F., Madeddu M., Pasciu V., Succu S., Spezzigu A., Satta V., Mereu P., Leoni G.G., Naitana S. (2009). Semen molecular and cellular features: These parameters can reliably predict subsequent ART outcome in a goat model. Reprod. Biol. Endocrinol..

[23] Goodson S.G., Qiu Y., Sutton K.A., Xie G., Jia W., O’Brien D.A. (2012). Metabolic substrates exhibit differential effects on functional parameters of mouse sperm capacitation. Biol. Reprod..

[24] Tourmente M., Rowe M., Gonzalez-Barroso M.M., Rial E., Gomendio M., Roldan E.R. (2013). Postcopulatory sexual selection increases ATP content in rodent spermatozoa. Evolution.

[25] Tourmente M., Villar-Moya P., Varea-Sanchez M., Luque-Larena J.J., Rial E., Roldan E.R. (2015). Performance of rodent spermatozoa over time is enhanced by increased ATP concentrations: The role of sperm competition. Biol. Reprod..

[26] Travis A.J., Jorgez C.J., Merdiushev T., Jones B.H., Dess D.M., Diaz-Cueto L., Storey B.T., Kopf G.S., Moss S.B. (2001). Functional relationships between capacitation-dependent cell signaling and compartmentalized metabolic pathways in murine spermatozoa. J. Biol. Chem..

[27] Visconti P.E., Krapf D., de la Vega-Beltran J.L., Acevedo J.J., Darszon A. (2011). Ion channels, phosphorylation and mammalian sperm capacitation. Asian J. Androl..

[28] Cosson J., Kuester E., Traugott G. (2013). ATP: The sperm movement energizer. Adenosine Triphosphate: Chemical Properties, Biosynthesis and Functions in Cells.

[29] Ruiz-Pesini E., Díez-Sánchez C., López-Pérez M.J., Enríquez J.A. (2007). The role of the mitochondrion in sperm function: Is there a place for oxidative phosphorylation or is this a purely glycolytic process?. Curr. Top. Dev. Biol..

[30] Storey B.T. (2008). Mammalian sperm metabolism: Oxygen and sugar, friend and foe. Int. J. Dev. Biol..

[31] Gardner D.K., Leese H.J. (1990). Concentrations of nutrients in mouse oviduct fluid and their effects on embryo development and metabolism in vitro. J. Reprod. Fertil..

[32] Harris S.E., Gopichandran N., Picton H.M., Leese H.J., Orsi N.M. (2005). Nutrient concentrations in murine follicular fluid and the female reproductive tract. Theriogenology.

[33] Tourmente M., Varea-Sanchez M., Roldan E.R.S. (2019). Faster and more efficient swimming: Energy consumption of murine spermatozoa under sperm competition. Biol. Reprod..

[34] Sansegundo E., Tourmente M., Roldan E.R.S. (2022). Energy metabolism and hyperactivation of spermatozoa from three mouse species under capacitating conditions. Cells.

[35] Balbach M., Gervasi M.G., Hidalgo D.M., Visconti P.E., Levin L.R., Buck J. (2020). Metabolic changes in mouse sperm during capacitation. Biol. Reprod..

[36] Yang Q., Wen Y., Wang L., Peng Z., Yeerken R., Zhen L., Li P., Li X. (2020). Ca^2+^ ionophore A23187 inhibits ATP generation reducing mouse sperm motility and PKA-dependent phosphorylation. Tissue Cell.

[37] Ferreira J.J., Cassina A., Irigoyen P., Ford M., Pietroroia S., Peramsetty N., Radi R., Santi C.M., Sapiro R. (2021). Increased mitochondrial activity upon CatSper channel activation is required for mouse sperm capacitation. Redox Biol..

[38] Giaccagli M.M., Gomez-Elias M.D., Herzfeld J.D., Marin-Briggiler C.I., Cuasnicu P.S., Cohen D.J., Da Ros V.G. (2021). Capacitation-induced mitochondrial activity is required for sperm fertilizing ability in mice by modulating hyperactivation. Front. Cell Dev. Biol..

[39] Tourmente M., Sansegundo E., Rial E., Roldan E.R.S. (2022). Capacitation promotes a shift in energy metabolism in murine sperm. Front. Cell Dev. Biol..

[40] Brokaw C.J. (1966). Effects of increased viscosity on the movements of some invertebrate spermatozoa. J. Exp. Biol..

[41] Gibbons B.H., Gibbons I.R. (1972). Flagellar movement and adenosine triphosphatase activity in sea urchin sperm extracted with triton X-100. J. Cell Biol..

[42] Brokaw C.J. (1975). Effects of viscosity and ATP concentration on the movement of reactivated sea-urchin sperm flagella. J. Exp. Biol..

[43] Chen D.T.N., Heymann M., Fraden S., Nicastro D., Dogic Z. (2015). ATP Consumption of eukaryotic flagella measured at a single-cell level. Biophys. J..

[44] Visconti P.E., Bailey J.L., Moore G.D., Pan D., Olds-Clarke P., Kopf G.S. (1995). Capacitation of mouse spermatozoa. I. Correlation between the capacitation state and protein tyrosine phosphorylation. Development.

[45] Visconti P.E., Moore G.D., Bailey J.L., Leclerc P., Connors S.A., Pan D., Olds-Clarke P., Kopf G.S. (1995). Capacitation of mouse spermatozoa. II. Protein tyrosine phosphorilation and capacitation are regulated by a cAMP-dependent pathway. Development.

[46] Gomez Montoto L., Varea Sanchez M., Tourmente M., Martin-Coello J., Luque-Larena J.J., Gomendio M., Roldan E.R. (2011). Sperm competition differentially affects swimming velocity and size of spermatozoa from closely related muroid rodents: Head first. Reproduction.

[47] Hyakutake T., Suzuki H., Yamamoto S. (2015). Effect of non-Newtonian fluid properties on bovine sperm motility. J. Biomech..

[48] Quill T.A., Sugden S.A., Rossi K.L., Doolittle L.K., Hammer R.E., Garbers D.L. (2003). Hyperactivated sperm motility driven by CatSper2 is required for fertilization. Proc. Natl. Acad. Sci. USA.

[49] Hyakutake T., Mori K., Sato K. (2018). Effects of surrounding fluid on motility of hyperactivated bovine sperm. J. Biomech..

[50] Lee M.A., Kopf G.S., Storey B.T. (1987). Effects of phorbol esters and a diacylglycerol on the mouse sperm acrosome reaction induced by the zona pellucida. Biol. Reprod..

[51] Leyton L., Saling P. (1989). Evidence that aggregation of mouse sperm receptors by ZP3 triggers the acrosome reaction. J. Cell Biol..

[52] Fraser L.R., Herod J.E. (1990). Expression of capacitation-dependent changes in chlortetracycline fluorescence patterns in mouse spermatozoa requires a suitable glycolysable substrate. J. Reprod. Fertil..

[53] da Fonseca Junior A.M., Gaita V., Argumedo D.R., de Castro L.S., Losano J.D.A., Ferreira Leite R., Nichi M., Assumpcao M., de Araujo D.R., Neves A.A.R. (2020). Changes in fertilization medium viscosity using hyaluronic acid impact bull sperm motility and acrosome status. Reprod. Domest. Anim..

[54] Mukai C., Okuno M. (2004). Glycolysis plays a major role for adenosine triphosphate supplementation in mouse sperm flagellar movement. Biol. Reprod..

[55] Hidalgo D.M., Romarowski A., Gervasi M.G., Navarrete F., Balbach M., Salicioni A.M., Levin L.R., Buck J., Visconti P.E. (2020). Capacitation increases glucose consumption in murine sperm. Mol. Reprod. Dev..

[56] Magdanz V., Boryshpolets S., Ridzewski C., Eckel B., Reinhardt K. (2019). The motility-based swim-up technique separates bull sperm based on differences in metabolic rates and tail length. PLoS ONE.

[57] Roldan E.R.S. (2019). Sperm competition and the evolution of sperm form and function in mammals. Reprod. Domest. Anim..

[58] Teves M.E., Roldan E.R.S. (2022). Sperm bauplan and function and underlying processes of sperm formation and selection. Physiol. Rev..

[59] Parker G.A. (1970). Sperm Competition and its evolutionary consequences in the insects. Biol. Rev..

[60] Gomendio M., Martin-Coello J., Crespo C., Magana C., Roldan E.R. (2006). Sperm competition enhances functional capacity of mammalian spermatozoa. Proc. Natl. Acad. Sci. USA.

[61] Suzuki H., Nunome M., Kinoshita G., Aplin K.P., Vogel P., Kryukov A.P., Jin M.L., Han S.H., Maryanto I., Tsuchiya K. (2013). Evolutionary and dispersal history of Eurasian house mice *Mus musculus* clarified by more extensive geographic sampling of mitochondrial DNA. Heredity.

[62] Cazaux B., Catalan J., Justy F., Escude C., Desmarais E., Britton-Davidian J. (2013). Evolution of the structure and composition of house mouse satellite DNA sequences in the subgenus *Mus* (Rodentia: Muridea): A cytogenomic approach. Chromosoma.

[63] Gomez Montoto L., Magana C., Tourmente M., Martin-Coello J., Crespo C., Luque-Larena J.J., Gomendio M., Roldan E.R. (2011). Sperm competition, sperm numbers and sperm quality in muroid rodents. PLoS ONE.

[64] Varea-Sanchez M., Tourmente M., Bastir M., Roldan E.R. (2016). Unraveling the Sperm Bauplan: Relationships between sperm head morphology and sperm function in rodents. Biol. Reprod..

[65] Tourmente M., Villar-Moya P., Rial E., Roldan E.R. (2015). Differences in ATP generation via glycolysis and oxidative phosphorylation and relationships with sperm motility in mouse species. J. Biol. Chem..

[66] Ivic A., Onyeaka H., Girling A., Brewis I.A., Ola B., Hammadieh N., Papaioannou S., Barratt C.L. (2002). Critical evaluation of methylcellulose as an alternative medium in sperm migration tests. Hum. Reprod..

[67] Eamer L., Nosrati R., Vollmer M., Zini A., Sinton D. (2015). Microfluidic assessment of swimming media for motility-based sperm selection. Biomicrofluidics.

[68] Lee M., Park J.W., Kim D., Kwon H., Cho M.J., Lee E.J., Shin T.E., Kim D.K., Lee S., Byeun D.G. (2021). Viscous cervical environment-on-a-chip for selecting high-quality sperm from human semen. Biomedicines.

[69] Gonzalez-Abreu D., Garcia-Martinez S., Fernandez-Espin V., Romar R., Gadea J. (2017). Incubation of boar spermatozoa in viscous media by addition of methylcellulose improves sperm quality and penetration rates during in vitro fertilization. Theriogenology.

[70] Shi Q.X., Roldan E.R. (1995). Bicarbonate/CO_2_ is not required for zona pellucida- or progesterone-induced acrosomal exocytosis of mouse spermatozoa but is essential for capacitation. Biol. Reprod..

[71] Ward C.R., Storey B.T. (1984). Determination of the time course of capacitation in mouse spermatozoa using a chlortetracycline fluorescence assay. Dev. Biol..

