# Supplementary material for: Effect of High Viscosity on Energy Metabolism and Kinematics of Spermatozoa from Three Mouse Species Incubated under Capacitating Conditions
Source: Int J Mol Sci. 2022 Dec 3;23(23):15247. doi: 10.3390/ijms232315247 (PMC9737050; doi:10.3390/ijms232315247)
Supplement: Supplementary file 1 [file ijms-23-15247-s001.zip › ijms-2031584-supplementary.pdf]

Supplementary Material to

Effect of high viscosity on energy metabolism and kinematics of spermatozoa from three mouse species incubated under capacitating conditions

Ana Sanchez-Rodriguez[1], Ester Sansegundo[1], Maximiliano Tourmente[1,2,3], Eduardo R. S. Roldan[1,*]

[1] Departmento de Biodiversidad y Biología Evolutiva, Museo Nacional de Ciencias Naturales (CSIC), 28006 Madrid, Spain

[2] Centro de Biología Celular y Molecular, Facultad de Ciencias Exactas, Físicas y Naturales, Universidad Nacional de Córdoba (FCEFyN - UNC), Córdoba X5016GCA, Argentina

[3] Instituto de Investigaciones Biológicas y Tecnológicas, Consejo Nacional de Investigaciones Científicas y Técnicas (IIByT - CONICET, UNC), Córdoba X5016GCA, Argentina

Figure S1..Variations in sperm parameters of three mouse species (*M. musculus*, *M. spretus* and *M. spicilegus*) over time in mT-H, mT-H + PVP, mT-BH and mT-BH + PVP.

Figure S2. Principal component analysis based on swimming parameters in sperm of three mouse species (*M. musculus*, *M. spretus* and *M. spicilegus*) in two incubation conditions.

Figure S3. Changes in principal components of sperm kinetics (OSV, OTS 1 and OTS 2) over time in sperm incubated in non-capacitating or capacitating conditions in high viscosity.

Figure S4. Changes in amount of ATP per sperm over time in sperm incubated in non-capacitating or capacitating conditions in high viscosity.

Table S1. Weight of each parameter for each principal component and Pearson's correlation in two principal components of overall sperm velocity (OSV) and two principal components of overall trajectory of spermatozoa (OTS) for *M. musculus*.

Table S2. Weight of each parameter for each principal component and Pearson's correlation in two principal components of overall sperm velocity (OSV) and two principal components of overall trajectory of spermatozoa (OTS) for *M. spretus*.

Table S3. Weight of each parameter for each principal component and Pearson's correlation in two principal components of overall sperm velocity (OSV) and two principal components of overall trajectory of spermatozoa (OTS) for *M. spicilegus*.

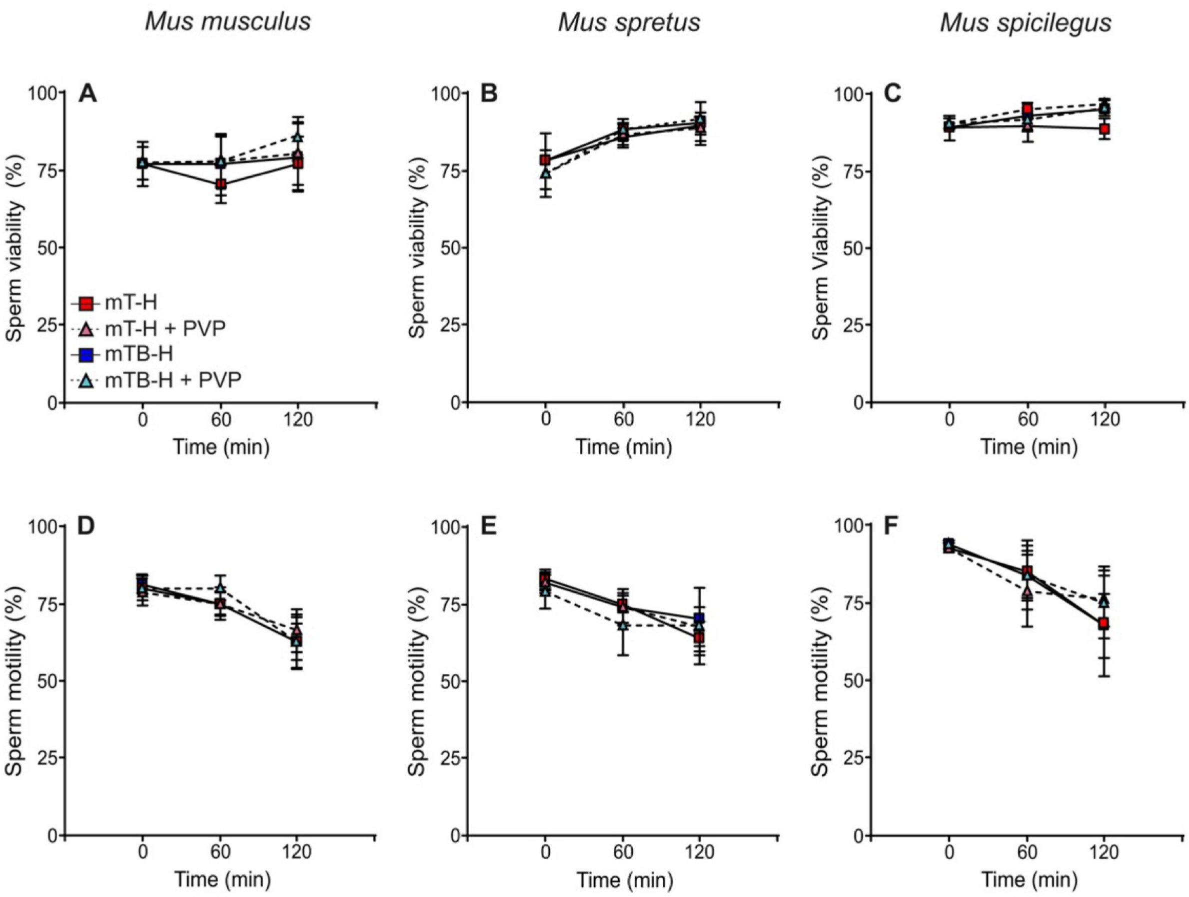

Figure S1..Variations in sperm parameters of three mouse species (*M. musculus*, *M. spretus* and *M. spicilegus*) over time in mT-H, mT-H + PVP, mT-BH and mT-BH + PVP. Data are means ± SE (*M. musculus*: N=4; *M. spretus*: N=5; *M. spicilegus*: N=4). A-C: Percentage of sperm viability. D-F: Percentage of sperm motility. G-I: Percentage of B pattern in CTC staining. A, D, G: *M. musculus*. B, E, H: *M. spretus*. C, F, I: *M. spicilegus*. Red squares: non-capacitating conditions with low viscosity, mT-H medium under air. Pink triangles: non-capacitating conditions with high viscosity, mT-H + PVP medium under air. Dark blue squares: capacitating conditions with low viscosity, mT-BH medium under 5 % CO_2/air. Light blue triangles: capacitating conditions with high viscosity, mT-BH + PVP under 5 % CO_2/air. There were no significant differences in any of the two response variables (motility and viability) in response to either incubation conditions or viscosity.

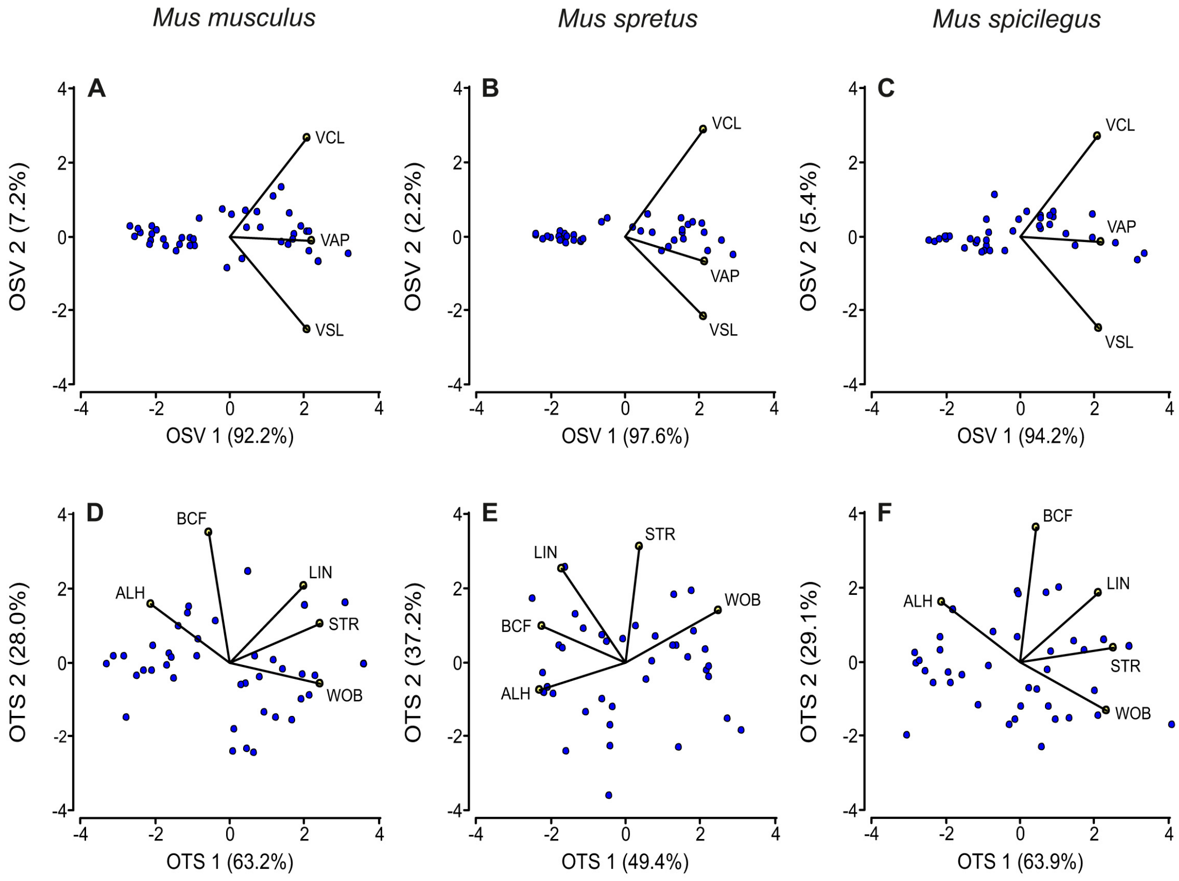

Figure S2. Principal component analysis based on swimming parameters in sperm of three mouse species (*M. musculus*, *M. spretus* and *M. spicilegus*) in two incubation conditions. A-C: Principal components of overall sperm velocity (OSV) of an analysis including curvilinear velocity (VCL), straight-line velocity (VSL) and average path velocity (VAP). The X-axis represents the value of first principal component of overall sperm velocity (OSV 1) and the Y-axis represents the value of second principal component of overall sperm velocity (OSV 2). D-F: Principal components of overall trajectory shape (OTS) of an analysis including linearity (LIN), straightness (STR), wobble coefficient (WOB), amplitude of lateral head displacement (ALH) and beat cross frequency (BCF). The X-axis represents the value of first principal component of overall trajectory shape (OTS 1) and Y-axis represents the second principal component of overall trajectory shape (OTS 2). A, D: *M. musculus*. B, E: *M. spretus*. C, F: *M. spicilegus*.

Table S1. Weight of each parameter for each principal component (VCL: curvilinear velocity; VSL: straight-line velocity, VAP: average path velocity; LIN: linearity; STR: straightness; WOB: wobble coefficient; ALH: amplitude of lateral head; BCF: beat cross frequency) and Pearson's correlation in two principal components of overall sperm velocity (OSV) and two principal components of overall trajectory of spermatozoa (OTS) for *M. musculus*. Results highlighted in bold show significant differences in statistical analysis ($p < 0.05$).

	OSV1		OSV2		OTS1		OTS2	
Parameter	weight	r_{xy}	weight	r_{xy}	wheight	r_{xy}	wheight	r_{xy}
VCL	**0.56**	**0,94**	**0.73**	**0,34**				
VSL	**0.57**	**0,95**	**-0.69**	**-0,32**				
VAP	**0.60**	**0,99**	-0.03	-0,02				
LIN					**0.47**	**0,78**	**0.39**	**0,54**
STR					**0.54**	**0,99**	0.16	0,27
WOB					**0.52**	**0,95**	-0.19	-0,15
ALH					**-0.47**	**-0,83**	**0.30**	**0,41**
BCF					-0.04	-0,22	**0.83**	**0,92**

Table S2. Weight of each parameter for each principal component (VCL: curvilinear velocity; VSL: straight-line velocity, VAP: average path velocity; LIN: linearity; STR: straightness; WOB: wobble coefficient; ALH: amplitude of lateral head; BCF: beat cross frequency) and Pearson's correlation in two principal components of overall sperm velocity (OSV) and two principal components of overall trajectory of spermatozoa (OTS) for *M. spretus*. Results highlighted in bold show significant differences in statistical analysis ($p < 0.05$).

	OSV1		OSV2		OTS1		OTS2	
Parameter	weight	r_{xy}	weigh t	r_{xy}	weight	r_{xy}	weight	r_{xy}
VCL	**0.57**	**0,98**	0.78	0,98				
VSL	**0.58**	**0,02**	-0.59	1,00				
VAP	**0.58**	**0,002**	-0.18	1,00				
LIN					**-0.38**	**-0,60**	**0.57**	**0,78**
STR					0.08	0,13	**0.71**	**0,96**
WOB					**0.56**	**0,89**	**0.31**	**0,43**
ALH					**-0.52**	**-0,82**	-0.17	-0,24
BCF					**-0.51**	**-0,80**	**0.22**	**0,29**

Table S3. Weight of each parameter for each principal component (VCL: curvilinear velocity; VSL: straight-line velocity, VAP: average path velocity; LIN: linearity; STR: straightness; WOB: wobble coefficient; ALH: amplitude of lateral head; BCF: beat cross frequency) and Pearson's correlation in two principal components of overall sperm velocity (OSV) and two principal components of overall trajectory of spermatozoa (OTS) for *Mus spicilegus*. Results highlighted in bold show significant differences in statistical analysis ($p < 0.05$).

	OSV1		OSV2		OTS1		OTS2	
Parameter	weight	r_{xy}	weight	r_{xy}	weight	r_{xy}	weight	r_{xy}
VCL	**0.57**	**0,94**	**0.73**	**0,94**				
VSL	**0.57**	**0,05**	-0.68	1,00				
VAP	**0.59**	**0,003**	-0.04	1,00				
LIN					**0.47**	**0,83**	**0.37**	**0,49**
STR					**0.54**	**0,98**	0.05	0,09
WOB					**0.50**	**0,91**	**-0.31**	**-0,35**
ALH					**-0.45**	**-0,83**	**0.37**	**0,42**
BCF					0.15	0,18	**0.79**	**0,95**

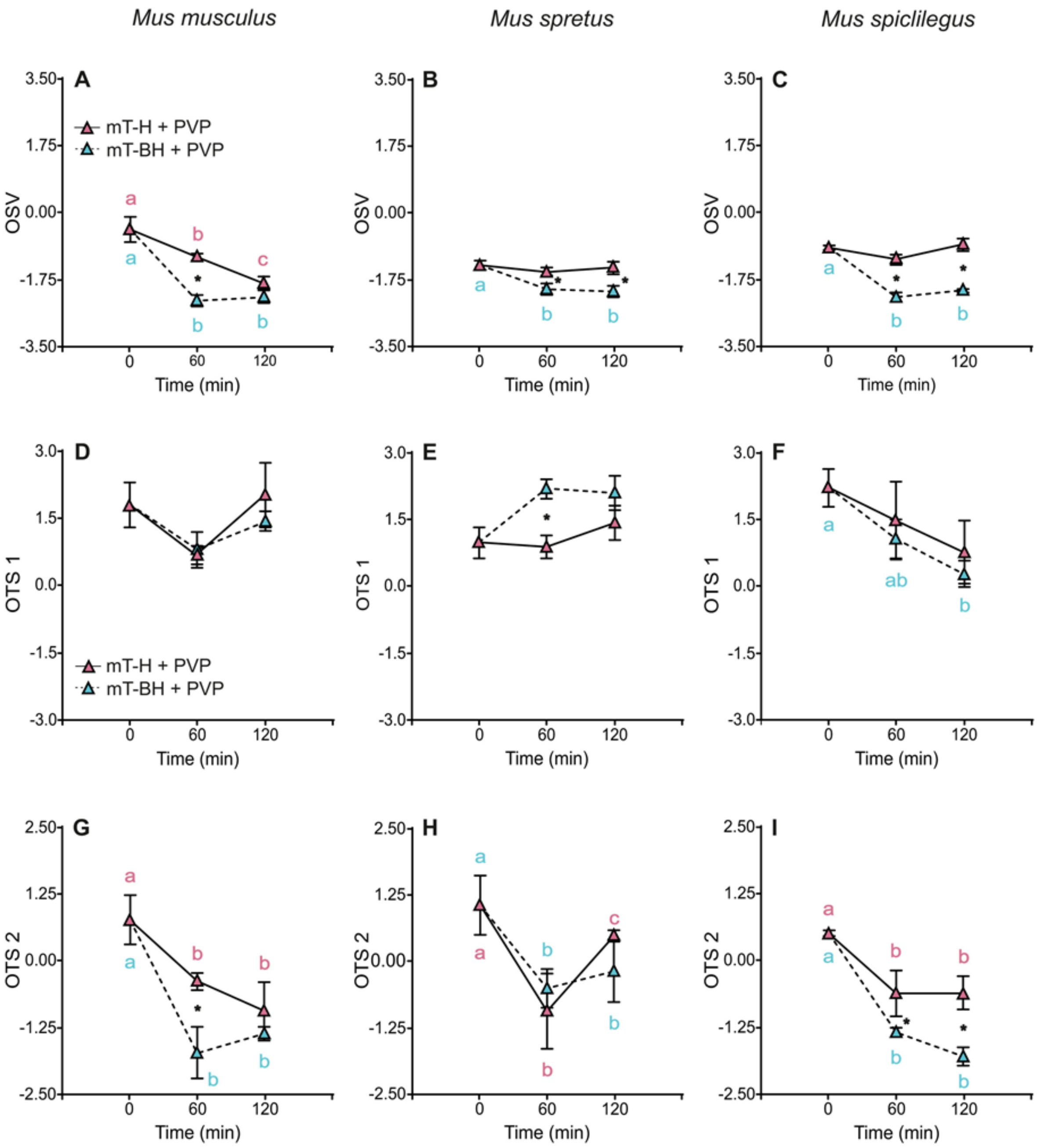

Figure S3. Changes in principal components of sperm kinetics (OSV, OTS 1 and OTS 2) over time in sperm incubated in non-capacitating or capacitating conditions in high viscosity. For non-capacitating conditions, spermatozoa were incubated in mT-H medium under air whereas for capacitating conditions sperm were incubated in mT-BH medium under 5% CO_2/air. OSV, overall sperm velocity; OTS 1 overall trajectory shape factor 1; OTS 2, overall trajectory shape factor 2. Data are means ± SE (*M. musculus*: N=4; *M. spretus*: N=5; *M. spicilegus*: N=4). A-C: OSV. D-F: OTS 1. G-H: OTS 2. A, D, G: *M. musculus*. B, E, H: *M. spretus*. C, F, I: *M. spicilegus*. Pink triangles: non-capacitating conditions in high viscosity (mT-H + PVP). Light blue triangles: capacitating conditions in high viscosity (mT-BH + PVP). Asterisks indicate significant differences between incubation media for the same time point in a Fisher *post-hoc* test ($p < 0.05$). Different letters indicate significant differences between time points for the same incubation medium in a Fisher *post-hoc* test ($p < 0.05$).

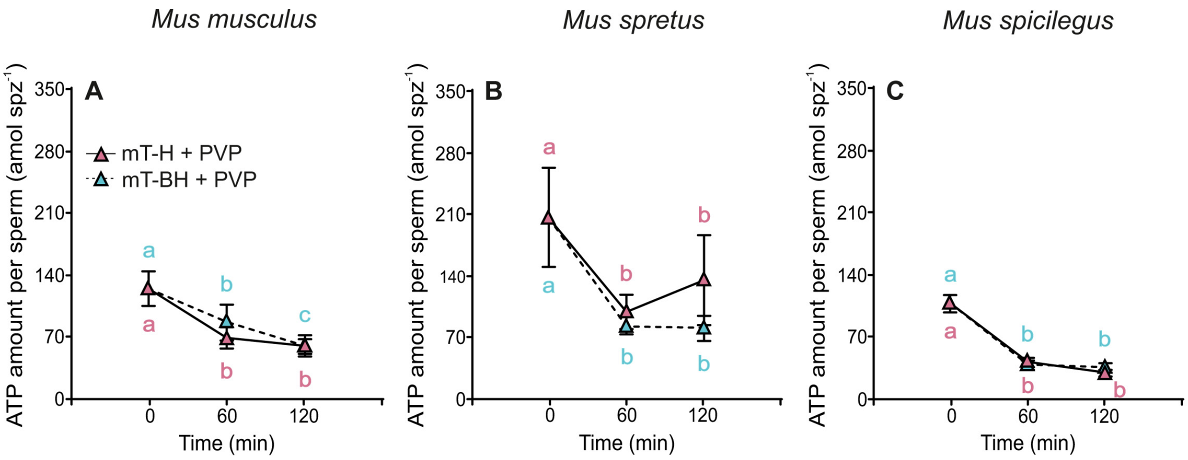

Figure S4. Changes in amount of ATP per sperm over time in sperm incubated in non-capacitating or capacitating conditions in high viscosity. For non-capacitating conditions, spermatozoa were incubated in mT-H medium under air whereas for capacitating conditions sperm were incubated in mT-BH medium under 5% CO_2/air. Data are means ± SE (*M. musculus*: N=4; *M. spretus*: N=5; *M. spicilegus*: N=4). A: *M. musculus*. B: *M. spretus*. C: *M. spicilegus*. Pink triangles: non-capacitating conditions in high viscosity (mT-H + PVP). Light blue triangles: capacitating conditions in high viscosity (mT-BH + PVP). Different letters indicate significant differences between time points for the same incubation medium in a Fisher *post-hoc* test ($p < 0.05$).
